# Identifying foetal forebrain interneurons as a target for monogenic autism risk factors and the polygenic *16p11.2* microdeletion

**DOI:** 10.1186/s12868-022-00771-3

**Published:** 2023-01-19

**Authors:** Yifei Yang, Sam A. Booker, James M. Clegg, Idoia Quintana-Urzainqui, Anna Sumera, Zrinko Kozic, Owen Dando, Sandra Martin Lorenzo, Yann Herault, Peter C. Kind, David J. Price, Thomas Pratt

**Affiliations:** 1 Simons Initiative for the Developing Brain, The University of Edinburgh, 15 George Square, Edinburgh, EH8 9XD United Kingdom; 2 Centre for Discovery Brain Sciences, The University of Edinburgh, 15 George Square, Edinburgh, EH8 9XD United Kingdom; 3 CNRS, Université de Strasbourg, INSERM, Institut de Génétique et de Biologie Moléculaire et Cellulaire, IGBMC, 1 rue Laurent Fries, 67404 Illkirch, France; 4 Department of Brain Sciences, Imperial College London, London, W12 0NN United Kingdom; 5 Developmental Biology Unit, European Molecular Biology Laboratory (EMBL), Meyerhofstrasse 1, 69012 Heidelberg, Germany

**Keywords:** Development, Telencephalon, Autism, Genetics, Single cell transcriptomics, GABAergic, Human, Rat, Electrophysiology, AIS

## Abstract

**Background:**

Autism spectrum condition or ‘autism’ is associated with numerous genetic risk factors including the polygenic *16p11.2* microdeletion. The balance between excitatory and inhibitory neurons in the cerebral cortex is hypothesised to be critical for the aetiology of autism making improved understanding of how risk factors impact on the development of these cells an important area of research. In the current study we aim to combine bioinformatics analysis of human foetal cerebral cortex gene expression data with anatomical and electrophysiological analysis of a *16p11.2*^*+/-*^ rat model to investigate how genetic risk factors impact on inhibitory neuron development.

**Methods:**

We performed bioinformatics analysis of single cell transcriptomes from gestational week (GW) 8–26 human foetal prefrontal cortex and anatomical and electrophysiological analysis of *16p11.2*^+/-^ rat cerebral cortex and hippocampus at post-natal day (P) 21.

**Results:**

We identified a subset of human interneurons (INs) first appearing at GW23 with enriched expression of a large fraction of risk factor transcripts including those expressed from the *16p11.2* locus. This suggests the hypothesis that these foetal INs are vulnerable to mutations causing autism. We investigated this in a rat model of the *16p11.2* microdeletion. We found no change in the numbers or position of either excitatory or inhibitory neurons in the somatosensory cortex or CA1 of *16p11.2*^*+/-*^ rats but found that CA1 Sst INs were hyperexcitable with an enlarged axon initial segment, which was not the case for CA1 pyramidal cells.

**Limitations:**

The human foetal gene expression data was acquired from cerebral cortex between gestational week (GW) 8 to 26. We cannot draw inferences about potential vulnerabilities to genetic autism risk factors for cells not present in the developing cerebral cortex at these stages. The analysis *16p11.2*^*+/-*^ rat phenotypes reported in the current study was restricted to 3-week old (P21) animals around the time of weaning and to a single interneuron cell-type while in human *16p11.2* microdeletion carriers symptoms likely involve multiple cell types and manifest in the first few years of life and on into adulthood.

**Conclusions:**

We have identified developing interneurons in human foetal cerebral cortex as potentially vulnerable to monogenic autism risk factors and the *16p11.2* microdeletion and report interneuron phenotypes in post-natal *16p11.2*^*+/-*^ rats.

**Supplementary Information:**

The online version contains supplementary material available at 10.1186/s12868-022-00771-3.

## Background

Autism spectrum conditions (ASC—referred to here as ‘autism’) describe several symptoms and behaviours which affect the way in which a group of people understand and react to the world around them (Mental Health Foundation) and may co-occur with other conditions including epilepsy and intellectual disability (ID). Recent efforts to understand the genetic landscape of autism identified hundreds of genetic risk factors predisposing to autism including de novo and inherited single gene mutation, copy number variations (CNVs) and chromosome anomalies although most cases have no known genetic cause [17, 25].

Genetic risk factors can be either ‘monogenic’ where a single gene mutation is sufficient to predispose to autism or ‘polygenic’ where one mutation directly affects several genes simultaneously. CNVs are an example of the latter where chromosomal microduplication or microdeletion affect the gene dosage of multiple genes. The de novo and inherited recurring 574 kb *16p11.2* CNV spans 27 protein coding genes on human chromosome 16. The *16p11.2* CNV is linked to several conditions collectively termed ‘16p11.2 syndrome’ with the microdeletion predisposing to autism, epilepsy, macrocephally, and obesity [38, 49, 80, 81].

Perhaps unsurprisingly given the genetic and symptomatic complexity of *16p11.2* syndrome, studies using human and animal models have identified diverse *16p11.2* neuronal phenotypes at various stages of development including differential gene expression, proliferation, signalling, cell and tissue anatomy, electrophysiology, and behaviour [1, 2, 6, 9, 19, 22, 35, 45, 55, 56, 59, 60, 65, 70, 76]. The *16p11.2* locus spans 27 protein coding genes each of which is a potential risk factor for the facets of *16p11.2* syndrome. Reduced dosage of individual *16p11.2* genes including *ALDOA*, *KCTD13*, *MAPK3*, *TAOK2, and MVP* have each been reported to impact neuronal proliferation and/or differentiation and the extent to which simultaneously reduced dosage of *16p11.2* genes synergise to produce microdeletion phenotypes is not fully understood [7, 21, 24, 30, 37, 54, 63, 83].

Autism manifests in early infancy and then persists into later life. A number of lines of evidence suggest that events during brain development in utero contribute to the subsequent development of symptoms [51]. The developing cerebral cortex is comprised of three neuronal cardinal cell classes (Neural progenitor cells (NPCs), excitatory neurons (ExNs), and inhibitory neurons (INs)) and three non-neuronal cardinal cell classes (astrocytes, oligodendrocyte precursors, and microglia). One important factor for cerebral cortex development is generating the functional balance between ExNs, which originate from NPCs located in the ventricular zone of the cerebral cortex, and INs which originate from NPCs located in the ganglionic eminence (GE) and then migrate into the cortex and integrate within functional circuits of the cortical plate [33, 41]. Changes in the number, position, anatomy, or electrophysiology of inhibitory or excitatory neurons may perturb the excitatory/inhibitory balance (the E/I balance) and is hypothesised to be a convergent mechanism in autism and its co-occuring conditions [1, 13, 47, 57, 61, 64].

The aim of the current study is to systematically identify types of cell in developing human cerebral cortex that are potentially vulnerable to autism risk factors using a single cell mRNA sequencing (scRNA-seq) dataset acquired from developing human foetal cortex spanning gestational weeks (GW) 8 to 26 [85]. While we found that some autism associated transcripts are differentially expressed between the cardinal cell classes our most striking observation was that a subset of differentiating INs first appearing at GW23 exhibited enriched expression of a number of monogenic and *16p11.2* risk factor transcripts. Gene ontology analysis indicated that these cells were likely to be undergoing cytoskeletal and synaptic differentiation suggesting that the *16p11.2* microdeletion may target IN differentiation in the foetal cortex contributing to IN phenotypes postnatally. Consistent with this idea we identified hypersensitive electrophysiology and enlarged axon initial segment (AIS) phenotypes in somatostatin (Sst) expressing hippocampal INs in a *16p11.2* microdeletion rat model.

## Methods

### Datasets

Three published cortical transcriptome datasets were used in this study to explore the gene expression pattern of autism-associated genes during cortical development.

**Fig. 1:**
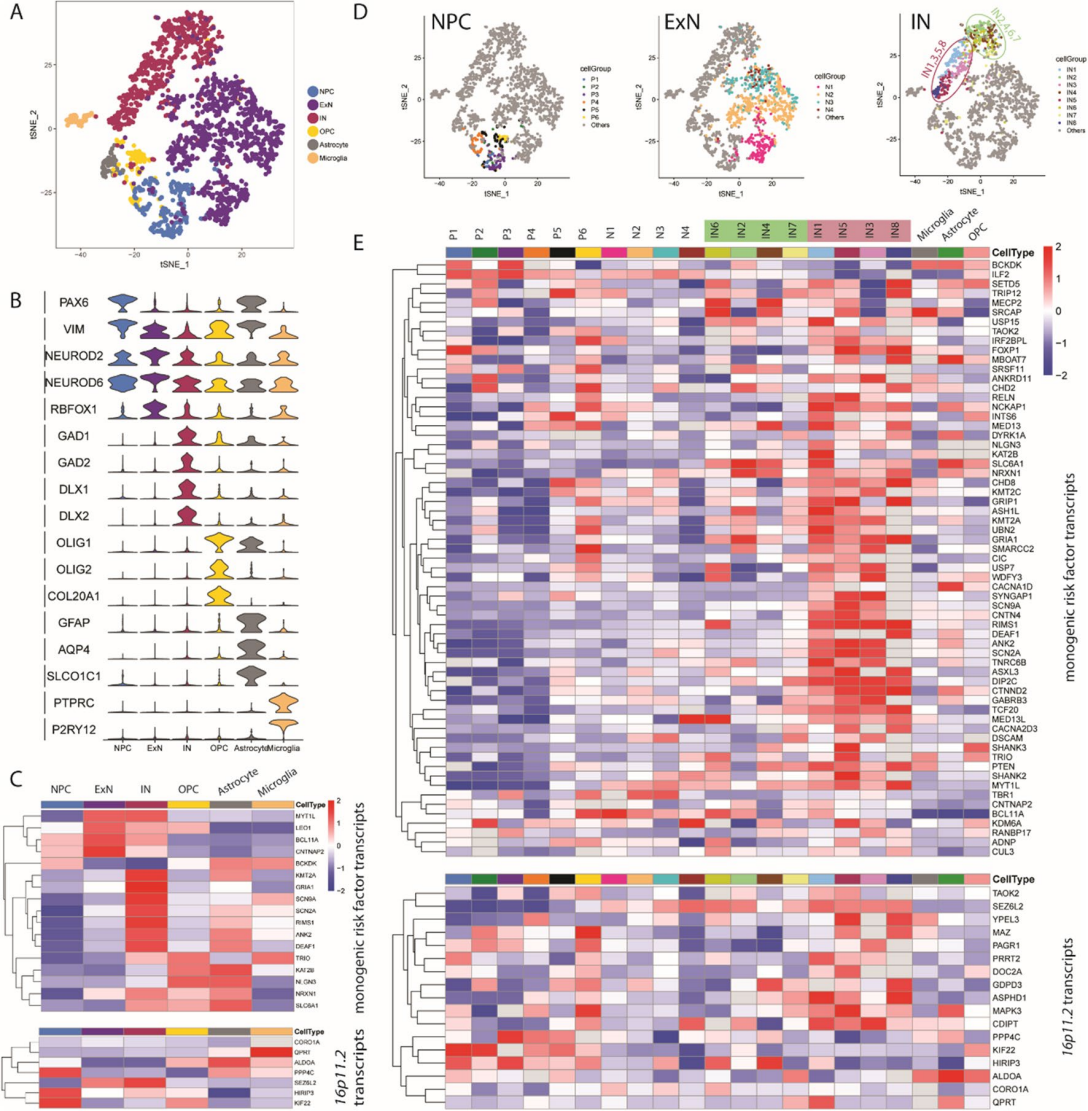
**A**–**C** Differential expression of autism risk factor transcripts among foetal cortical cardinal cell classes. **A** *t*-SNE plot showing the cardinal cell classes identified in the dataset. **B** Violin plot illustrating the expression pattern of marker genes among the six cardinal cell classes. **C** Heatmap illustrating the expression pattern of significantly differentially expressed autism risk factor transcripts across cardinal cell classes (Wilcox test, adjust p < 0.05, log (fold change) > 0.3). Top: monogenic autism risk factor transcripts; Bottom: *16p11.2* transcripts. **D**, **E** Unsupervised clustering within the cardinal classes and similarity comparison between cell clusters. **D** Unsupervised clustering subdividing the cardinal classes into 21 different cell clusters. OPC, astrocytes, and microglia were not further clustered. **E** Heatmap illustrating the expression pattern of differentially expressed autism risk factor transcripts across 21 cell clusters (Wilcox test, adjust p < 0.05, log (fold change) > 0.3) for differentially expressed monogenic autism risk factor transcripts (top panel) and differentially expressed *16p11.2* transcripts (bottom panel)

The raw gene expression matrix in the scRNA-seq data of human foetal PFC was obtained from the Gene Expression Omnibus (GEO) under the accession number GSE104276, then the data was normalized as the original paper described [85]. We used the authors’ original classification of 6 cardinal cell classes (NPC, ExN, IN, OPC, Astrocyte and Microglia) Fig. 1.

The expression matrix of genes in the adult human cortical single nuclei RNA-seq data were downloaded under the accession number of GSE97930 [39]. In the dataset from Lake et al., only cells that identified as “INs” were used for further analysis. The original 8 interneuron clusters were grouped based on the expression pattern of marker genes (In1/2/3 as VIP, In4 as NG, In6 as PV, In7/8 as SST, Fig. 2B, C in [39].

**Fig. 2:**
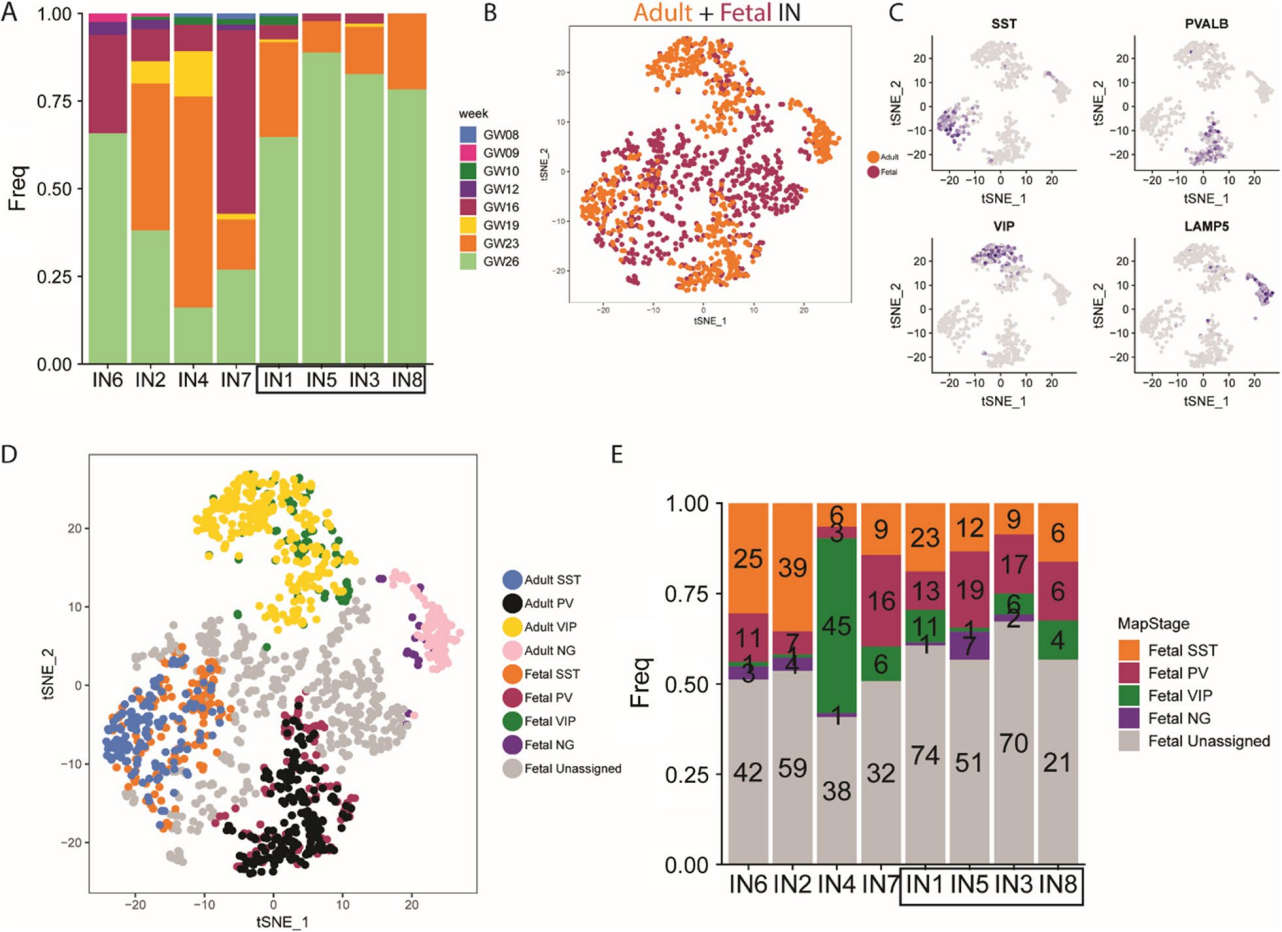
**A** Bar plot depicting the proportion of sample ages in each IN cluster. **B** Canonical correspondence analysis (CCA) integrating the foetal and adult human scRNA-seq data. **C** Gradient plots showing gene expression pattern of marker genes of IN lineages in t-SNE space. **D** CCA-KNN analysis in t-SNE space provides a method to categorise immature INs into SST, PV, VIP and Neuroglia form (NG) classes according to their transcriptomic similarity to mature neurons from human adult cortex. **E** Bar plot depicting the number and proportion of IN cell types in each IN cluster

For the mouse scRNA-seq datasets of *Dlx6a-cre* fate-mapped cortical inhibitory neurons, the pre-processed Seurat objects were downloaded from the author’s share link (https://www.dropbox.com/s/qe2carqnf9eu4sd/Filtered_Mayer-et-al.Rda.zip?dl=0) [43]. We used the authors’ original classification of seven IN cell types (Sst, Pvalb, Vip, Id2, Nos1, Th and Igfbp6).

All 3 datasets were converted into Seurat objects by R package Seurat (version 2.3.0) for further analysis. In detail, in the dataset from Zhong et al., raw read counts were normalized based on the original paper described. Any cells with less than 1000 genes expressed were removed, and any gene expressed by less than 3 cells at less than 1 normalized expression value was removed. Pseudogenes, miRNA, rRNA, mitochondrial associated and ribosome related genes were excluded from further analysis. The filtered gene expression matrix and the classification of the cardinal cell classes were used to create a Seurat object. We also create a Seurat object for the dataset from Lake et al. using the same procedure. The pre-processed Seurat object from Mayer et al. was not changed. The scRNA-Seq data was also analyzed with BBrowser version 2.2.44 (SingleCell).

### Lists of autism risk genes

Monogenic autism associated genes were downloaded from the SFARI database (released May 2019) (https://gene.sfari.org/database/human-gene/) and the 83 highest ranking (SFARI 1 + 2) were analysed as these genes are significant statistically in genome-wide studies between cases and controls. Besides these monogenetic genes, the copy number variance (CNV) of genetic loci (CNV genes), either deletions or duplications, are also linked to autism. We selected the 27 protein coding genes at the *16p11.2* locus since both duplication and deletion of these genes has been linked to significantly increased incidence of autism representing a potentially polygenic cause of autism.

### Clustering and visualization of cell types

The identification of six cardinal cell classes were obtained from the original paper and re-plotted in a two-dimensional space of t-Distributed Stochastic Neighbor Embedding (tSNE). In details, the highly variable genes (HVGs) were identified using Seurat function FindVariableGenes. The mean of logged expression values was plotted against variance to mean expression level ratio (VMR) for each gene. Genes with log transformed mean expression level between 1 and 8, and VMR lower than 1.2 were considered as highly variable genes. Then principal component analysis (PCA) was performed with RunPCA function in Seurat using HVGs to analyze all the cells. Following the PCA, we conducted JACKSTRAW analysis with 100 iterations to identify statistically significant (p value < 0.01) principal components (PCs) that were driving systematic variation. We used tSNE to present data in two-dimensional coordinates, generated by RunTSNE function in Seurat, and the first 7 significant PCs identified by JACKSTRAW analysis were used as input to RunTSNE function. Perplexity was set to 20. t-SNE plot and the violin plot were generated using R package ggplot2.

We further clustered the three cardinal cell classes (NPC, ExN and IN) from the foetal cortical dataset. Due to the different number of cells and the variant gene expression pattern in each cardinal cell class, the HVGs were identified using the same method but with the different parameters. For the cells in NPC, genes with log transformed mean expression level between 0.5 and 8, and VMR lower than 1.2 were considered as highly variable genes. For the cells in ExN and IN classes, genes with log transformed mean expression level between 1 and 10, and VMR lower than 0.5 were considered as highly variable genes. Then the statistically significant PCs were calculated by JACKSTRAW analysis and used as input to get tSNE coordinates. Clustering was done with Luvain Jaccard algorithm using t-SNE coordinates by FindClusters function from Seurat. The resolution parameters used to IDENTIFY clusters within the three cardinal cell classes were: NPC, resolution = 1; ExN, resolution = 0.1; and IN, resolution = 0.5. Other parameters that we left at default.

### Comparison of IN clustering

To compare the Zhong’s and Yang’s IN clustering results, two figures were plotted to illustrate the comparison. Firstly, the alluvial plot was draw by geom_alluvium funcion in ggplot2 R package (Additional file 1: Figure S1C). Secondly, the top 3000 highly variable genes (HVGs) were calculated by FindVariableFeatures function in Seurat R package, then the average log-transformed expression of these HVGs across all clusters were calculated. The Pearson correlation was performed to demonstrate the clusters-clusters similarity between two clustering results, and the correlation was further plotted as the heatmaps by heatmap.2 function in gplots R package (Additional file 1: Figure S1D) [27].

### Identification of differential expressed genes

All differential expression (DE) analyses were conducted using Seurat function *FindAllMarkers*. In brief, we took one group of cells and compared it with the rest of the cells, using Wilcoxon rank sum test. For any given comparison we only considered genes that were expressed by at least 33% of cells in either population. Genes that exhibit p values under 0.05, as well as log fold change over 0.33 were considered significant. All heatmaps of DE analysis were plotted using R package pheatmap (Fig. 1D, E).

### MetaNeighbor analysis

MetaNeighbor analysis was performed using the R function MetaNeighbor with default settings [20]. The AUROC (Area under the Receiver Operating Characteristic) scores produced by MetaNeighbor analysis indicate the degree of correlation between cell clusters. Three gene lists were used as input to do MetaNeighbor analysis among the 21 clusters of human foetal dataset: Highly variable genes (HVGs) identified as significant differentialy expressed genes (DEGs) between the clusters (Additional file 1: Figure S2A); monogenic autism risk genes (Additional file 1: Figure S2B); and*16p11.2* genes (Additional file 1: Figure S2C). The results from the MetaNeighbor analysis were plotted as a heatmap using the gplots function heatmap. For a given gene set each pairwise comparison between cell clusters is given an AUROC score ranging from 1.0 (red on the heatmap) indicating that cells were highly probable to be of the same type to 0.0 (blue on the heatmap) indicating that it was highly improbable that the cells were of the same type. A score on 0.5 (yellow on the heatmap) indicates that the gene set used was unable to distinguish between the cells better than by chance.

### Construction of IN pseudotime trajectory

**Fig. 3:**
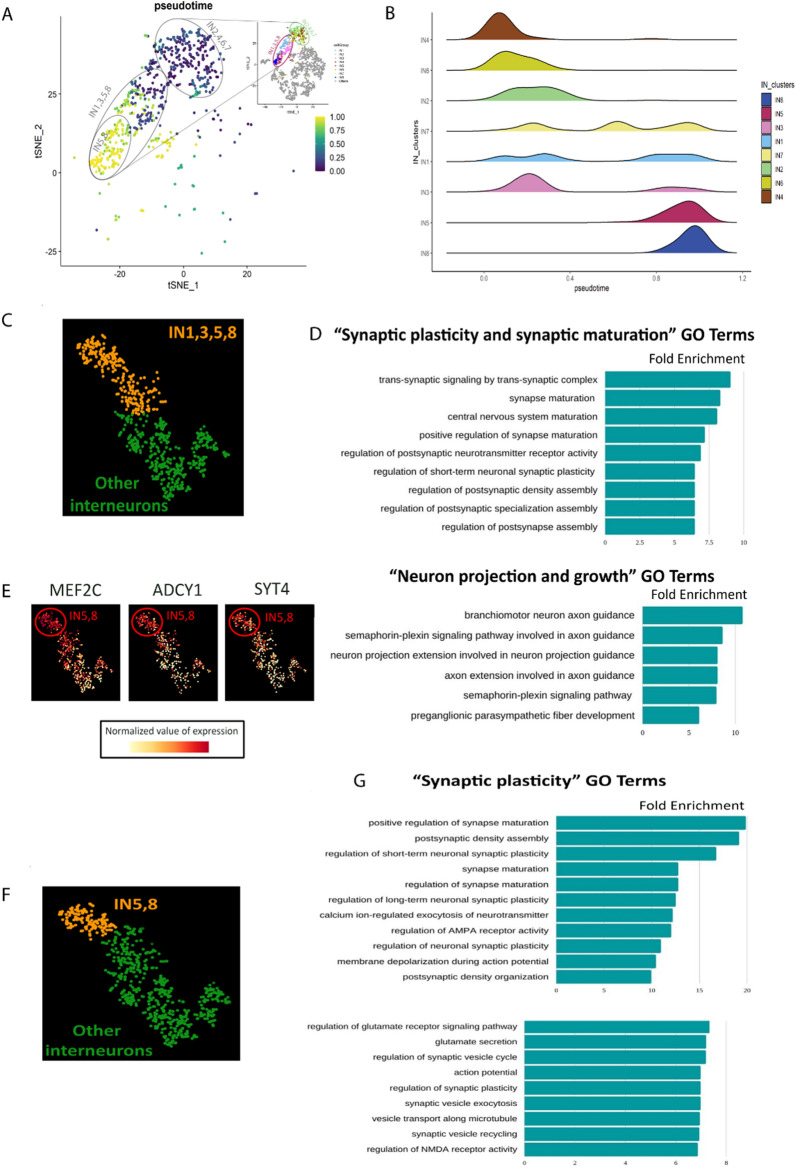
Characterisation of INs by pseudotime trajectory and gene ontology analysis. **A**, **B** pseudotime analysis. **A** t-SNE plot showing the developmental trajectory of interneurons with inferred pseudotime trajectories by Monocle3 (inset reproduced from Fig. 1D for reference). Each dot represents an individual cell in IN 1–8 and coloured according to it’s position on the pseudotime trajectory from 0.00 (least differentiated—dark blue) to 1.00 (most differentiated—yellow). **B** Density plot estimating the distribution of numbers of cells in each interneuron cluster (IN 1–8) along the pseudotime trajectory. **C**–**G** Gene ontology (GO) analysis. GO analysis in IN1,3,5,8 (orange in **C**) versus other INs (green in **C**) reveals enrichment of GO terms associated with **D** synaptic maturation and plasticity and axon extension and guidance. **E** gradient plots of *MEF2C*, *ADCY1*, and *SYT4* showing that these transcripts are expressed in a gradient across INs with highest expression in IN5,8. **F**, **G** Gene ontology (GO) analysis in IN5,8 (orange in **F**) versus other INs (green in **F**) reveals enrichment of GO terms associated with **G** synaptic maturation and plasticity

The developmental trajectory of human cortical INs was constructed with R package Monocle3 with default parameters [14, 58, 75]. The Seurat object of INs was extracted from the whole human dataset, and the object was converted to a SingleCellExperiment object. Then this SingleCellExperiment object was re-normalized and pre-processed by the preprocess_cds function in Monocle3. After pre-processing, the function reduce_dimension were applied for dimension reduction, and the trajectory graph was built by learn_graph function. Finally, cells were ordered along pseudotime trajectory with the function of order_cells in Monocle3. The R package ggplot2 was used to plot the pseudotime information on the tSNE coordinates of INs as shown in Fig. 1D, as well as the density plot shown in Fig. 3A. To infer the dynamic gene expression of *MEF2C*, *ADCY1*, *SYT4* and *CAMK2* (Fig. 3E and Additional file 1: Fig S4G) we constructed a gene expression plot along the pseudotime using the geom_smooth function in ggplot2 R package with LOWESS smoothing model (Additional file 1: Figure S4D).

### Gene ontology analysis

The resulting gene list, ordered by sign-adjusted P value, was the input for gene set enrichment analysis to test for enriched gene ontology (GO) terms using the clusterProfiler package version 3.4.4 with default settings. GO term analysis was performed on three categories (Biological process. Molecular function. Cellular component), and gene sets with a BH adjusted P < 0.05 were considered to be significantly enriched. The top three significant GO terms in each category were plotted by R package ggplot2.

### Projection based on multiple datasets

We conducted canonical correlation analysis (CCA) and *k*-nearest neighbors analysis (KNN) as we previous described to classify the cell types of foetal INs based on the cell type features in the adult transcriptomics datasets [44]. Briefly, we first performed random forest analysis within HVGs to do feature selection for both foetal and adult human cortical INs. Then we selected the shared HVGs between two datasets that best represented the feature of IN cell types. The HVGs were used as input gene list to RunCCA function, and the first 4 dimensions were used as input to AlignSubspace function. The aligned projection vectors were used as input to do dimensional reduction by RunTSNE function. Perplexity was set to 40. We used the two t-SNE coordinates for adult cells to conduct KNN and re-assign foetal IN identities using the knn.cv function from R package FNN. A foetal IN was assigned the identity represented by the majority, and at least 5, of its closest 30 neighbours; in case of ties, the cell remains unassigned. t-SNE plots, and the bar plots were generated using R package ggplot2.

### Animals

All rats were bred in-house according to Home Office UK legislation and licenses approved by the University of Edinburgh Ethical Review Committees and Home Office. Animal husbandry was in accordance with UK Animals (Scientific Procedures) Act of 1986 regulations. Rat 16p11.2 DEL rat model (*16p11.2*^*+/-*^*)* was generated by CRISPR/Cas9 genome editing of the Sprague Dawley line [59]. Rats were maintained on the Sprague Dawley background and genotyped by PCR as previously described [59]. P21 rat tissue was fixed by transcardial perfusion with 4% paraformaldehyde in PBS, brains were then dissected and immersed in 4% paraformaldehyde in PBS overnight at 4 °C.

### In Situ hybridisation and immunofluorescence labelling

Brains were cryoprotected in 30% sucrose in PBS, embedded in OCT and sectioned at a thickness of 10 µm using a cryostat (Leica, CM3050 S). Frozen sections were then mounted on SuperFrost Plus™ slides (Thermo Fisher). *Gad1* In situ hybridisation on frozen sections was performed as previously described [79]. NeuN Immunofluorescence was performed following in situ hybridisation as described previously [18] with rabbit anti-NeuN (1/300, Abcam) Secondary antibodies used were donkey anti-goat Alexa Fluor 488 and donkey anti-rabbit Alexa Fluor 568 (both used 1/200 and from Life Technologies). Tissue was counterstained using DAPI (1/1000, Life Technologies).

Axon initial segment (AIS) labelling, was performed as previously described [50]. Briefly, rats were perfused as described above, then post-fixed for 1 h at room temperature with 4% paraformaldehyde in 0.1 M phosphate buffer (PB). Brains were then transferred to 0.1 M phosphate buffered saline (PBS) and 60 µm thick coronal slices containing the CA1 region of hippocampus were cut on an oscillating blade vibratome (Leica VT1000, Leica, Germany) and transferred to PBS. Briefly, sections were rinsed in PBS then transferred to a blocking solution containing 10% normal goat serum, 0.3% Triton X-100 and 0.05% NaN_3_ diluted in PBS for 1 h. Primary antibodies raised against AnkyrinG (1:1000; 75–146, NeuroMab, USA) and somatostatin (Somatostatin-14, T-4102.0400; 1:1000, Peninsula Labs, USA) were applied in PBS containing 5% normal goat serum, 0.3% Triton X-100 and 0.05% NaN_3_ for 24–72 h at 4 °C. Slices were washed with PBS and then secondary antibodies applied (Goat anti-rabbit AlexaFluor 488 and Goat anti-mouse AlexaFluor 633, Invitrogen, UK, both 1:500) in PBS with 3% normal goat serum, 0.1% Triton X-100 and 0.05% NaN3 added, overnight at 4 °C. Sections were then washed with PBS, desalted in PB, and mounted on glass slides with Vectashield® mounting medium (Vector Labs, UK). Confocal image stacks of either the *str. pyramidale* or *str. oriens/*alveus border were acquired on a Zeiss LSM800 laser scanning microscope equipped with a 63x (1.4 NA) objective lens at 1024 × 1024 resolution (step size of 0.25 µm). Individual AIS were measured offline using ImageJ as segmented lines covering the full extent of AnkyrinG labelling observed. As in SSt INs the AIS often emerges from a proximal dendrite, they were only identified where they emerged from a clearly fluorescent labelled dendrite (see Additional file 1: Figure S8). A minimum of 25 AIS were measured from each rat.

For identification of SST INs, slices were fixed following whole-cell patch-clamp recording (see below) and fixed overnight in 4% PFA in 0.1 M PB. Immunofluorescent labelling was performed according to the same protocol as above, but excluding the AnkyrinG antibody. Secondary antibodies (goat anti-rabbit AlexaFluor488, 1:500, Invitrogen, Dunfermline, UK) were applied with the added inclusion of fluorescent-conjugated streptavidin (Streptavidin AlexaFluor 633, 1:500, Invitrogen, Dunfermline, UK) to visualise recorded neurons. We observed somatic labelling for SST consistent with our previous research [10].

### Imaging

All fluorescence images were acquired using either a Leica AF6000 epifluorescence microscope coupled to a Leica DFC360 digital camera running Leica LAS-X software, or a Nikon Ti: E Inverted confocal microscope running Nikon NIS-Elements Confocal software.

### NeuN/Gad1 cell quantification

*Gad1*^+^ and NeuN^+^ cells within the cortex were quantified by counting *Gad1*^+^ (red) and NeuN^+^ cells (green) within a 200 µm wide column spanning the somatosensory cortex (indicated region, Fig. 5A). *Gad1*^+^ and NeuN^+^ cell position was quantified by counting cells in 10 adjacent counting bins within the same 200 µm wide column spanning the somatosensory cortex.

*Gad1*^+^ and NeuN^+^ cells within the hippocampus were quantified by counting cells within the *str. oriens* and *str. pyramidale* of the CA1 region (indicated region, Fig. 5A). To control for the varying size of the counting area *Gad1*^+^ cell number was expressed as *Gad1*^+^ cells per length (mm) of the CA1 region, length was measured along the centre of the *str. pyramidale*. Gad1^+^ cells were classified as belonging to *str. pyramidale* if in contact with NeuN^+^;*Gad1*^*−*^ pyramidal cells, all other hippocampal *Gad1*^+^ cells superficial to this layer were classified as belonging to *str. oriens.* All measurements and quantification was performed using FIJI software.

### In vitro slice electrophysiology

Acute rat brain slices were prepared as previously described [50]. Briefly, rats were decapitated without anaesthesia and the brain rapidly dissected into ice-cold sucrose-modified artificial cerebrospinal fluid (ACSF; in mM: 87 NaCl, 2.5 KCl, 25 NaHCO_3_, 1.25 NaH_2_PO_4_, 25 glucose, 75 sucrose, 7 MgCl_2_, 0.5 CaCl_2_), which was saturated with carbogen (95% O2/5% CO2). 400 μm horizontal brain slices were cut on a vibratome (VT1200S, Leica, Germany) and transferred to sucrose-ACSF at 35 °C for 30 min and then room temperature until needed.

For whole-cell patch-clamp recordings slices were transferred to a submerged recording chamber flowing with pre-warmed ACSF (in mM: 125 NaCl, 2.5 KCl, 25 NaHCO_3_, 1.25 NaH_2_PO_4_, 25 glucose, 1 MgCl_2_, 2 CaCl_2_), bubbled with carbogen, and perfused a rate of 4–6 mL.min^−1^ at 30 ± 1 °C). Slices were viewed under infrared differential inference contrast microscopy with a digital camera (SciCamPro, Scientifica, UK) mounted on an upright microscope (SliceScope, Scientifica, UK) with 40 × water-immersion objective lens (1.0 N.A., Olympus, Japan). Recording pipettes were pulled from borosilicate glass capillaries (1.7 mm outer/1 mm inner diameter, Harvard Apparatus, UK) on a horizontal electrode puller (P-97, Sutter Instruments, CA, USA), which when filled with a K-gluconate based internal solution (in mM 142 K-gluconate, 4 KCl, 0.5 EGTA, 10 HEPES, 2 MgCl_2_, 2 Na_2_ATP, 0.3 Na_2_GTP, 1 Na_2_Phosphocreatine, 2.7 Biocytin, pH = 7.4, 290–310 mOsm) which resulted in a 3–5 MΩ tip resistance. Cells were rejected if: they were more depolarised than -50 mV, had series resistance > 30 MΩ, or the series resistance changed by more than 20% during the recording. Recordings were performed with a MultiClamp 700B (Molecular Devices, CA, USA) amplifier and filtered online at 10 kHz with the built-in 4-pole Bessel filter and digitized at 20 kHz (Digidata1550B, Molecular Devices, CA, USA).)

Cells were identified either as CA1 pyramidal cells (CA1 PCs) with having large, ovoid somata located in *str. pyramidale* and an apical dendrite entering *str. radiatum* or somatostatin INs having bipolar, horizontally oriented somata at the *str. oriens*/alveus border. All intrinsic membrane properties were measured in current-clamp. Passive membrane properties, included resting membrane potential, membrane time constant, and input resistance, were measured from hyperpolarising steps (− 10 pA, 500 ms duration), from resting membrane potential. Active properties were determined from a series of hyper- to depolarising current steps (− 500 to + 500 pA, 500 ms) from a holding potential of − 70 mV, maintained with a bias current injection. All AP properties were determined from the first AP elicited above rheobase. Spontaneous EPSCs were measured in voltage-clamp from a holding potential of − 70 mV and detected offline based on a triexponential curve fit and a threshold of 3*SD of the baseline noise. Traces were collected in pCLAMP 9 (Molecular Devices, CA, USA) and stored on a desktop computer. Analysis of electrophysiological data was performed offline Stimfit [32], blind to both genotype. All data from somatostatin INs is shown only for those cells where clear immunofluorescent labelling was detected at the level of the soma.

### Statistics

All rat experiments and analyses were performed blind to genotype, which were sampled in a random manner between experimental days. All data shown as mean ± standard error of the mean (SEM), with the number of cells (n) and animals (N) indicated where appropriate. All electrophysiology data are reported as cell averages. All histology data (AIS lengths and cell counts) are shown as animal averages. Minimum sample size was calculated based on our previous effect size for cellular hyperexcitability and AIS length [10, 11], assuming 80% power to determine 95% probability of rejecting the null-hypothesis. Statistical comparisons were performed using a linear mixed-effect model (or its generalised form) using the *lme4* package in R [5], with genotype or cell-type as fixed effect, with slice/animal/litter included as random effects. Based on the linear mixed-effects model, p-values for statistical effects were tested using the Wald test, based on effect size and variance determined from the relevant mixed-effects model. For experiments examining the density of interneurons and principal cells, animal average densities were the principal replicate which was tested with 2-way ANOVA. Statistical significance was assumed if p < 0.05.

## Results

### Differential expression of autism risk genes among foetal cortical cardinal cell classes

A general supposition is that functional disruption of a gene will more likely affect the cells expressing high levels of it’s transcript. Based on this principle, a cell type expressing high levels of an autism risk factor transcript is regarded as potentially vulnerable to genetic mutation in the corresponding gene with the resulting cellular phenotype contributing to the development of autism. Accordingly, we have calculated differential expression of autism associated transcripts among cell types in foetal human cerebral cortex to identify cells potentially vulnerable to autism genetic risk factors during brain development.

We started with a scRNA-seq dataset comprising 2306 cells taken from human foetal pre-frontal cortex spanning gestational weeks (GW) 8 to 26 [85]. At these stages the cerebral cortex contains neural progenitor cells (NPCs) destined to become ExNs, ExNs, and INs which have migrated into the cerebral cortex. This dataset does not include NPCs destined to become INs as these reside in the GE. The six cardinal cell types were identified in the authors’ original classification comprising neural (NPC, ExN, and IN) and non-neural (oligodendrocyte precursor cell (OPC), astrocyte, and microglia) cells (Fig. 1A, B and Additional file 1: Fig. S1A, B). Differentially expressed genes (DEGs) were calculated across these 6 cardinal cell classes. Based on the DEGs, we find that the six cell classes showed distinct cardinal class aggregation and specific gene expression profiles. A list of well-known cell class markers that are included in the DEGs was used to illustrate the classification across six cardinal cell classes [48, 53] (Fig. 1B). The markers used to identify different cardinal cell classes were: *PAX6*, *HES2* and *VIM* (NPCs); *NEUROD2*, *NEUROD6* and *RBFOX1* (ExNs); *GAD1*, *GAD2*, *DLX1* and *DLX2* (INs); *OLIG1*, *OLIG2* and *COL20A1* (OPCs); *GFAP*, *AQP4* and *SLCO1C1* (astrocytes); *PTPRC* and *P2RY12* (microglia). The expression pattern of these marker genes show that the cardinal cell classes were correctly represented in our analysis (Fig. 1B).

Then we identified the expression pattern of autism risk factor transcripts across the cardinal cell classes and found that 17/83 high confidence and strong candidate monogenic risk factor transcripts and 7/27 *16p11.2* transcripts were significantly differentially expressed (Wilcox test, adjust p < 0.05, log (fold change) > 0.3) between cardinal cells classes (Fig. 1C). A heatmap of expression of the monogenic autism risk factor transcripts (Fig. 1C—top) and the *16p11.2* transcripts (Fig. 1C—bottom) shows expression of each autism risk factor transcript (rows) in each of the six cardinal cell classes (columns). Transcript levels with expression greater than average across the cardinal classes are shown in red, while transcripts with lower than average expression are shown in blue. There was no obvious pattern to suggest that any cardinal class was particularly vulnerable to a large proportion of either monogenic autism genetic risk factors or the *16p11.2* microdeletion.

### Identification of human foetal INs potentially vulnerable to genetic autism risk factors

The single-cell approach allows us to investigate the variability of highly expressed genes among molecularly defined cell subpopulations and identify cells within cardinal classes which may be vulnerable to genetic autism risk factors. Based on unsupervised clustering, we subdivided the cardinal classes into 21 different cell clusters (Fig. 1D): 6 for NPCs (P1-6); 4 for ExNs (N1-4); and 8 for INs (IN1-8). The non-neuronal cardinal cell classes (OPC, astrocytes, and microglia) contained small numbers of tightly clustered cells and were not further subdivided. The transcriptional relationships between IN clusters identified by [85] and in the current study using the same scRNAseq data are illustrated in Additional file 1: Fig S1C, D.

We hierarchically clustered the 21 clusters according to transcriptomic similarity using MetaNeighbour analysis with ~ 2000 highly variable genes (Additional file 1: Fig. S2A) and used this ordering to generate a heatmap of the 62/83 monogenic risk factor transcripts (Fig. 1E, top) and 17/27 *16p11.2* transcripts genes (Fig. 1E, bottom) that were significantly (Wilcox test, adjust p < 0.05, log (fold change) > 0.3) differentially expressed between clusters. Violin plots of all autism risk transcripts (including those not differentially expressed between clusters) for the 83 monogenic autism risk transcripts (Additional file 1: Fig. S3A) and 27 *16p11.2* transcripts (Additional file 1: Fig. S3B) show the expression profile in each cluster.

Of the differentially expressed monogenic risk factor transcripts (Fig. 1E, top) a few were enriched in NPCs, (for example *IRF26PL*, *BCL11A*, and *CHD2*), with fewer transcripts enriched in ExNs (for example *TBR1*). Other transcripts were expressed across many clusters (for example *KMT2A, ILF2, SMARCC2, SRSF11, UPF3B, TNRC6B*), while others showed relatively low expression across cell types (for example *GRIN2B, MAGEL2, and MET*). A striking feature of the heatmap was a preponderance of relatively high gene expression (red shading) in the IN cell-types (for example *SCN2A*, *SCN9A, DEAF1, SHANK2, RIMS1, GRIP1, SYNGAP1*), which was most apparent for the IN subgroups IN1,3,5,8 (purple highlight in Fig. 1E and circle in Fig. 1D) compared to IN2,4,6 7 (green highlight in Fig. 1E and circle in Fig. 1D) and other clusters.

We observed a similar pattern for *16p11.2* transcript expression (Fig. 1E, bottom) with a preponderance of high expression in IN clusters1, 3, 5, 8, indicating specifically enriched expression of subset of *16p11.2* transcripts (for example *SEZL6L2*, *PRRT2*, *QPRT*, and *YPEL3*) with the next highest number of enriched transcripts in progenitor clusters (for example *KIFF22* and *PPC4C*). Other transcripts are expressed both in progenitors and INs but much less in excitatory neurons (for example *MAPK3*), and others broadly across all cell clusters (for example *TMEM219*) or at very low levels in any cluster (*ASPHD1*, *C16orf54*, *C16orf92*, *SPN*, *TBX6*, *ZG16*).

To investigate how well the expression of autism risk factor transcripts defined the cell clusters more systematically we used MetaNeighbor analysis which reports on how similar cells are to each other based on expression of a given input gene set using AUROC scores [20]. From this, we generated a pairwise comparison matrix between the 21 cell clusters (Additional file 1: Fig. S2). Performing MetaNeighbor with the same ~ 2000 DEGs used to perform hierarchical clustering (Additional file 1: Fig. S2A) we confirmed that cells in each cardinal class were generally more similar within class (red on heatmap) and less similar (blue on heatmap) between cardinal classes. Nevertheless, some ExNs (N1 and N2) were quite similar to progenitors (P1-P6) likely indicating that they represented relatively immature excitatory neurons that retained some progenitor identity. Within the INs there was a clear divide between IN1, 3, 5, 8 (red box in Additional file 1: Fig. S2A–C) and IN2, 4, 6 (green box in Additional file 1: Fig. S2A–C) cluster groups, with highest similarity within group and low similarity between groups. Next, we performed the same analysis for the 83 SFARI monogenic autism risk factor transcripts (Additional file 1: Fig. S2B) and found that strongest similarity was retained within the progenitor group P1-P6 and the IN group IN1,3,5,8. A similar pattern emerged when we used the 27 *16p11.2* transcripts as the gene-set (Additional file 1: Fig. S2C), although here the strongest similarity was within the NPC P1-6 and between IN5 and IN8. These results indicate that the immature INs can be robustly distinguished from other cells in the developing brain by their expression pattern of autism risk factor transcripts.

We conclude from the gene expression analysis that a subset of developing INs, IN1,3, 5, 8, in GW8-26 human foetal cerebral cortex express a high proportion of autism associated transcripts at higher levels than other cells. This suggests that IN1,3,5,8 represent INs that have migrated into the cerebral cortex and that are vulnerable to a large number of monogenic and the*16p11.2* microdeletion risk factors.

### Properties of human foetal INs potentially vulnerable to autism risk factors

Having identified IN1,5,3,8 as a potentially important class of INs we next examined their developmental and transcriptional properties. We found that IN1,3,5,8 were largely absent during the earlier stages (GW8-19) of cerebral cortex development and appear at GW23 with the vast majority of IN1,5,3,8 INs present at GW26 (Fig. 2A). On the other hand, IN2,4,6,7 clusters contained higher proportions of cells from earlier stages (Fig. 2A), suggesting that IN1,3,5,8 might represent a more mature state than the rest of IN clusters in our dataset. INs have reached the cortex in substantial numbers by GW16 (Additional file 1: Fig. S1B) indicating that IN1, 3, 5, 8 cells correspond to a stage of the developmental trajectory after tangentially migrating INs enter the cortex [33, 41].

We used canonical correlation analysis (CCA-KNN) to integrate foetal [85] and mature [39] human IN scRNA-seq datasets to identify mature cell types corresponding to IN1, 3, 5, 8 clusters. We first reduced the dimensionality of.

both datasets (adult IN cells = orange; foetal IN cells = blue) onto the same 2-dimensional space using *t*-SNE (Fig. 2B), which allowed the identification of 4 major cell types of adult INs based on the expression of variable genes shared between both datasets. We then assigned identities to adult based on expression of markers for PV, SST, VIP and neurogliaform cells illustrated by gradient plots of *SST*, *PVALB*, *VIP* and/or *LAMP5* transcripts to identify defined classes of cortical INs (Fig. 2C). This then allowed us to assign foetal INs to each of these cell types based on transcriptional similarity to the mature INs (Fig. 2D). Of the IN1, 3, 5, 8 cells classified in this manner we found that they were not homogenous, but rather consisted of PV, SST, and VIP cell types (Fig. 2E). A parsimonious interpretation is that the foetal IN1, 5, 3, 8 are cells destined to become several categories of mature IN cell types, although this awaits further investigation as assignation of cell lineage from scRNA-seq data is ambiguous.

The developing cerebral cortex contains INs at various stages of differentiation. To understand how our IN clusters map onto the IN developmental trajectory we first used gene expression to order the IN cells in pseudotime to give an indication of their differentiation state (Additional file 1: Fig. S4A). We then mapped their position on the pseudotime trajectory to the IN1-8 clusters revealing that the IN1,3,5,8 cells are overall more differentiated than the IN2,4,6,7 (Fig. 3A) with IN4,6 representing only the earliest stages of the pseudotime trajectory and IN5,8 only the latest (Fig. 3A, B and Additional file 1: Fig. S4 BC).

To gain further insight into the identity and developmental cell state represented by IN1,3,5,8, we performed differential gene expression analysis with respect to other INs (Fig. 3C) and used the transcripts enriched in IN1, 3, 5, 8 with a log fold change higher than 3 (1623 genes FDR < 0.001) to test for Gene Ontology (GO, biological process) enrichment. We found that within the top 30 GO terms (ordered according to Fold Enrichment), 9 categories were related to synaptic plasticity, synaptic maturation and synaptic transmission and 6 categories were related to neuron projection and growth (Fig. 3D), with fold enrichments ranging from 6 to 10, suggesting that IN1, 3, 5, 8 cells show earlier maturation of neurites and synapses than other INs. The Gene Ontology term “Regulation of Synaptic Plasticity” contains 192 genes, from which 54 are differentially expressed in IN1, 3, 5, 8. A closer inspection of the expression pattern in the t-SNE space showed that many of these genes followed a general expression level gradient trend with its maximum levels in INs corresponding to IN5 and IN8 (*MEF2C*, *ADCY1*, and *SYT4* shown as examples in Fig. 3E and Additional file 1: Fig S4D). This suggested that the INs in the dataset are ordered in the t-SNE space according to a gradient of synapse formation, with IN5,8 being the higher extreme of this axis. To confirm this, we performed differential expression analysis between IN5,8 IN cells versus all other INs (Fig. 3F) and found a high enrichment of synaptic plasticity-related terms but this time showing fold enrichments ranging from 7 to 20 (Fig. 3G), almost doubling the values of the previous comparison.

Finally, to gain further insight into IN5,8 neuronal identity, we compared IN5,8 cluster versus all other neurons (including both excitatory and inhibitory, Additional file 1: Fig. S4E). Interestingly, enriched functional terms were mainly related to synaptic plasticity, learning and social behaviour (Additional file 1: Fig. S4F). Visual inspection of gradient plots in the t-SNE space confirmed that many of the genes linked to synaptic plasticity and maturation are selectively expressed in IN5,8 (*CAMK2* and *ADCY1* are shown as examples in Additional file 1: Fig. S4G). Overall our pseudotime and GO analysis indicate that the IN cells which we identified as being vulnerable to autism genetic risk factors in the cerebral cortex at GW23-26 are comprised of the more differentiated INs elaborating processes and forming synapses.

This raises the possibility that by targeting the stage of the IN developmental trajectory represented by IN1, 3, 5, 8, genetic autism risk factors perturb the development of the physiological properties of foetal INs. The remaining interneurons IN2, 4, 6, 7 appear less vulnerable and represent an earlier stage in the IN developmental trajectory. Our analysis suggests genetic autism risk factors impact on all main IN lineages during foetal development and may affect their function into postnatal life.

### Conservation of potentially vulnerable INs between humans and rodents

These analyses of human foetal neurons suggest the hypothesis that a large proportion of autism related genes regulate IN development in the human foetal cortex. A prediction of this hypothesis is that there will be IN phenotypes initiated during human brain development in utero that persist into postnatal life and predispose to autism and its co-occurring conditions. Such investigation is currently not possible, however, rodent models provide a complementary means to test cellular vulnerability to autism genetic risk factors under physiological conditions. Rodent INs both at early stages of their development as NPCs in the GE and in adult cerebral cortex are molecularly similar to their human counterparts [4, 66]. We next confirmed that foetal and early post-natal rodent cerebral cortex contains INs with similar molecular properties to human foetal cerebral cortex INs IN1, 3, 5, 8 identified above.

We identified two mouse scRNAseq data sets comprising FACS sorted cortical INs at embryonic day (E) 18.5 and postnatal (P) day 20, when INs are differentiating and forming circuits [43]. Violin plots of *16p11.2* (Additional file 1: Fig. S5A) and monogenic autism risk factor (Additional file 1: Fig. S5B) transcript expression in mouse E18.5 and P20 cortical INs shows a high proportion of risk factor transcripts are detected and most are shared with human foetal cortical INs (compare Additional file 1: Fig. S3A, B to Fig. S3A, B) suggesting broad conservation of developing IN vulnerability to autism genetic risk factors. The *16p11.2* transcript *PAGR1* and some monogenic risk factor transcripts, for example *ANK2*, *BAZ2B*, *CUX1*, *DDX3X*, *FOXP1*, *MECP2*, *SCN2A*, *SYNGAP1*, *TNRC6B*, and *TRIO,* are detectable in human but not mouse INs. This most likely stems from technical differences, including sensitivity, between the mouse and human gene expression data but could reflect evolutionary divergence.

**Fig. 4:**
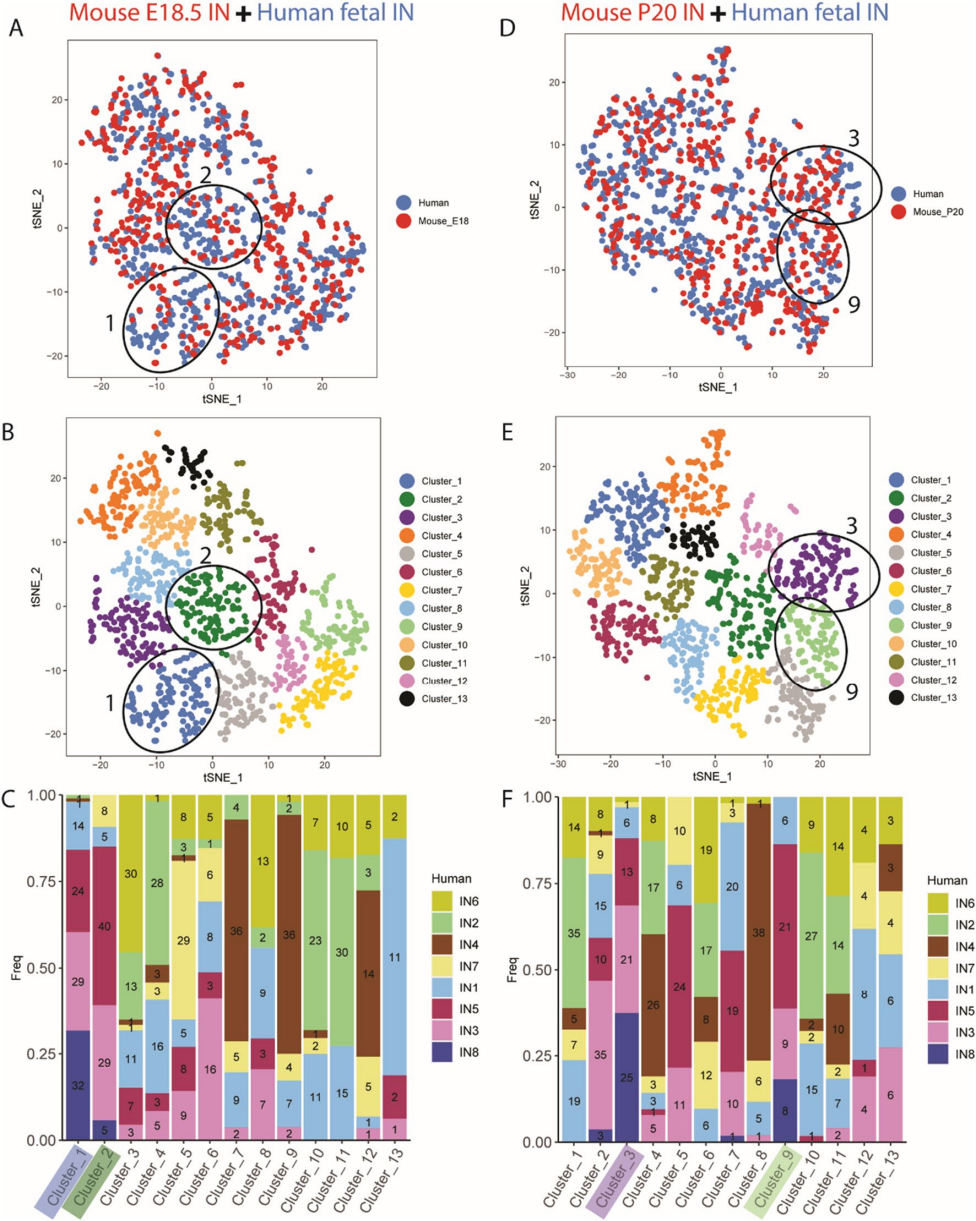
Identifying transcriptomic correlates between developing human and mouse INs at (**A**–**C**) E18.5 and (**D**–**F**) P20. **A**, **D** CCA integration of mouse (red) and human (blue) INs in tSNE space. (**B**, **B**)JACCARD clustering into 13 mixed clusters. **C**, **F** Distribution of human IN1-8 INs in each of the mixed clusters with numbers of cells shown within each bar. The mixed clusters 1&2 for E18.5 (**A**–**C**) and 3&9 for P20 (**D**–**F**) that are most enriched for human IN1,3,5,8 cells are indicated on each panel

For each mouse developmental stage we used CCA-KNN to integrate the mouse and human INs into the same tSNE space (Fig. 4A, D) to allow us to identify mixed clusters of transcriptomically similar mouse and human INs (Fig. 4B, E). For each mouse developmental stage, we examined how the human IN cell types IN1-8 were distributed between the mixed mouse + human clusters (Fig. 4C, F). This analysis revealed that E18.5 clusters 1 and 2 (indicated in Fig. 4A–C) and P20 clusters 3 and 9 (indicated in Fig. 4D–F) contained the greatest enrichment of human IN1,3,5,8 cells. Critically, these clusters contained comparable numbers of mouse and human cells indicating that the developing and postnatal mouse possesses INs molecularly similar to human IN1, 3, 5, 8 cells. Together these findings support the use of rodents as a valid model to investigate the impact of autism risk factors on developing INs.

### Changes to IN function in the rat model of 16p11.2 microdeletion

We next set out to investigate the impact of the *16p11.2* microdeletion by performing anatomical and electrophysiological characterisation in a rat *16p11.2* microdeletion model (*16p11.2*^±^ rats) at P21, a stage at which the rodent cortex contains INs molecularly similar to IN1,3,5,8 (Fig. 4 D-F) [59].

**Fig. 5:**
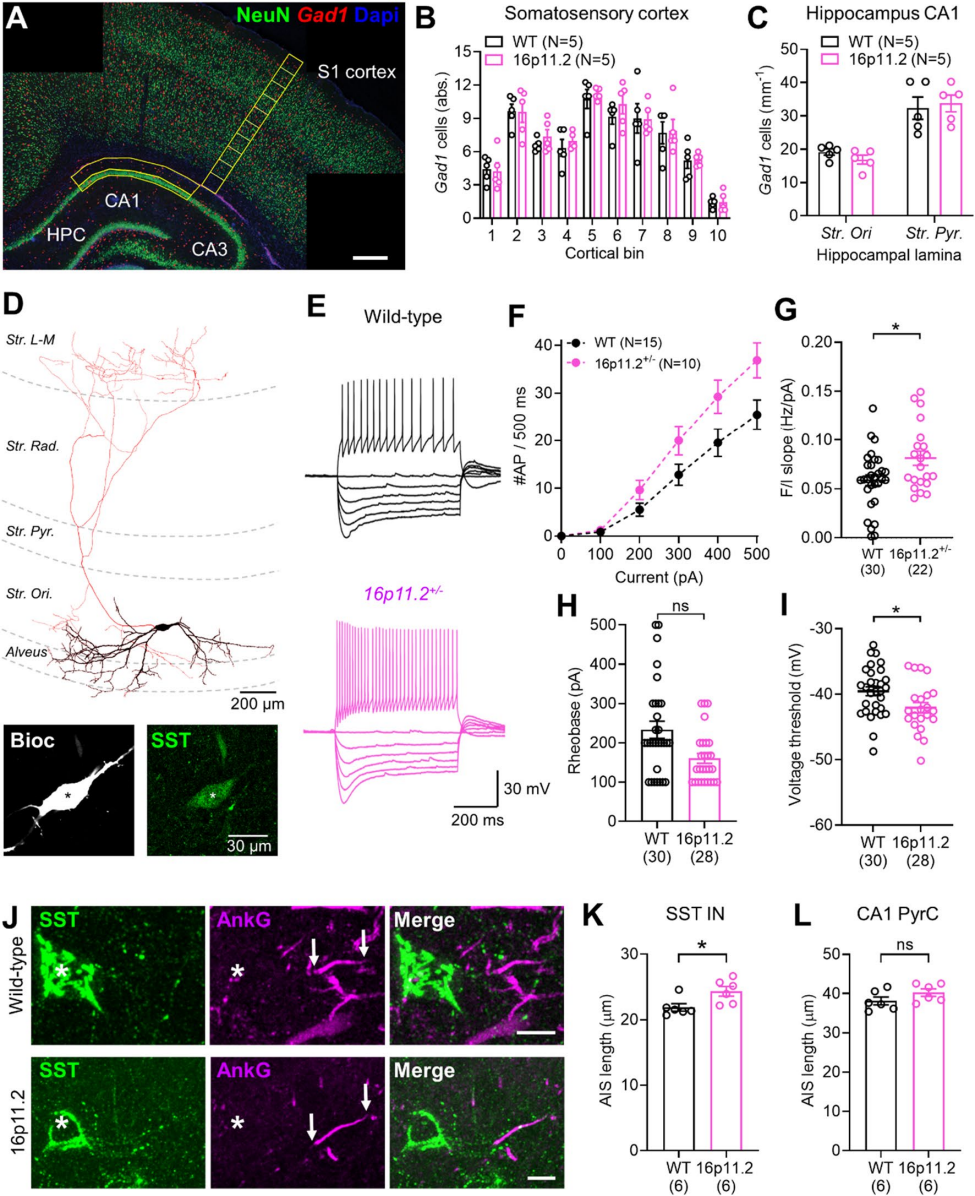
Selective hyperexcitability of SSt INs in *16p11.2*^+/-^ rat hippocampus, but with no change in IN number. **A** Overview micrograph of *Gad1* mRNA and NeuN protein expression in the rat hippocampus and cortex at P21. Gad1 expressing INs (red) and NeuN expressing, Gad1 negative excitatory neurons (green) can be observed in the cortex and hippocampus. Scale bars 400 µm **B** Quantification of *Gad1*-positive IN (IN) number through the somatosensory cortex in WT (black, n = 5) and *16p11.2*^+/-^ (pink, n = 5) rats. Counting areas indicated yellow in A with cortical bins numbered from 1 at the ventricular edge to 10 at the pial surface. **C** Quantification of the combined total number of *Gad1*-positive neurons in the s*tr.oriens* (*Str. ori*) and s*tr. pyramidale* (*Str pyr.*) of the CA1 region of the hippocampus in WT (N = 5) and 16p11.2^+/-^ (N = 5) rats. Counting area indicated in (**A**). **D** example reconstruction of a recorded CA1 SST IN. Dendrites are shown in black and axons shown in red. Hippocampal layers are indicated (dashed grey lines) with respect to the *alveus, Str. Ori, Str. Pyr, Str. Radiatum (Rad.), and Str. Lacunosum-moleculare* (*L-M*). **D**, inset immunoreactivity for SST (green) from the same cell shown in comparison to the biocytin filled soma. **E** Representative action potential discharge in response to hyper- to depolarising current steps in SST-INs, from the *str. oriens* of CA1 from WT (top) and *16p11.2*^+/-^ rats (bottom). **F** Summary current-frequency plots from identified SSt INs from WT (N = 15 rats) and *16p11.2*^+/-^ (N = 10 rats). **G** Measured slope of individual current-frequency (F/I) plots from all cells. **H** Quantification of rheobase current in identified SSt-INs from WT (n = 30 cells) and *16p11.2*^+/-^ (n = 28 cells) rats. (**I**) Measurement of the voltage threshold of the first action potential elicited at rheobase for the same cells in (**F**). **J** Representative micrographs showing immunohistochemical labelling for SSt (green), the AIS marker AnkyrinG (AnkG, magenta), and their overlap (merge). The SSt soma is indicated with an asterisk (*) and the start and end of the AIS localised to that IN indicated (arrows). Scale bar: 10 µm. **K** Quantification of the AIS length of SSt INs from WT (N = 6 rats, n = 162 AIS) and *16p11.2*^+/-^ (N = 6 rats, n = 151 AIS). **L** Quantification of AIS lengths of putative CA1 pyramidal cells from WT (N = 6 rats, n = 150 AIS) and *16p11.2*^+/-^ (N = 6 rats, n = 155 AIS) rats. Statistics shown: ns – p > 0.05, *—p < 0.05, from Linear Mixed Effects modelling

We first determined the whether the *16p11.2* microdeletion affects number of INs and ExNs populating the cortex in *16p11.2*^±^ rats, compared to WT littermates. To achieve this, we performed in situ hybridization for *Gad1*, in conjunction with immunohistological labelling for the pan-neuronal nuclear marker NeuN, to identify *Gad1*-negative putative excitatory neurons and *Gad1*-positive INs in the cortex (Fig. 5A). No change to the laminar positioning or density of INs in the somatosensory cortex was observed in *16p11.2*^±^ rats, compared to wild-type (WT) littermates (Fig. 5B), nor was any change to the total number (Fig. S6A) or proportion (Additional file 1: Fig. S6B) of INs detected within the somatosensory cortex. To confirm that this was not confined to the neocortex, we next examined IN number in hippocampal area CA1 (Additional file 1: Fig. S6C). Consistently, IN number in *str. oriens* (SO) and *st. pyramidale* (SP) of CA1 was unchanged between WT and *16p11.2*^+/-^ rats (*t*_(8)_ = -0.09, *p* = 0.93 *t* test, Additional file 1: Fig. S6D). Total IN number within the SO and SP was also unchanged indicating that the position of INs within the hippocampus is unaffected in *16p11.2*^±^ rats (Fig. 5C). Taken together, these data confirm that heterozygosity of *16p11.2* genes does not impact the final regional organisation of IN number or position within the neocortex or hippocampus.

Our analyses indicate that foetal human IN 1, 3, 5, 8 cells contribute to all the major IN classes including somatostatin (SST) expressing INs (Fig. 2E). We next asked whether the functional properties SST-INs might be impacted by the *16p11.2* microdeletion in our rat model. We chose to focus our investigation on SST-INs in CA1 because of their known roles in the aetiology of autism-associated phenotypes in rodent models (see Discussion) and experimental tractability. These SST IN share close homology to rodent cell types and originate from a common embryonic niche in the GE independent of cortical destination [3, 16]. To determine whether heterozygosity for *16p11.2* microdeletion directly impairs the function of SST INs independent of cell number we performed whole-cell patch-clamp recordings from identified SST INs from *16p11.2*^+/-^ rats and WT littermates.

SST-INs in CA1 of the hippocampus are highly-identifiable with horizontally organised somata located at the *str. oriens/alveus* border, with horizontally organised dendrites and typically with an axon extending into *str. lacunosum-moleculare* [12]. In the present study, we recorded a total 30 WT (from 15 rats) and 28 *16p11.2*^+/-^ (10 rats) SSt INs (Fig. 5D), all of which displayed clear immunoreactivity for SST at the somata (Fig. 5D, lower). In response to depolarising current injections (0–500 pA, 100 pA steps), SSt INs from WT rats generally responded with high-frequency action potential discharge (Fig. 5E), which had a peak action potential discharge of 27.4 ± 2.5 action potentials/500 ms (Fig. 5F). By comparison, we found that SSt INs.

from *16p11.2*^+/-^ rats displayed elevated action potential discharge, compared to WT controls both in terms of the frequency/current (F/I) curve (F_(5, 58)_ = 5.10, P = 0.0006 [genotype x current], N = 15 WT and 10 *16p11.2*^+/-^ rats, Fig. 5F), and also the overall F/I slope (t_(26)_ = 2.33, P = 0.028, Student’s unpaired t-test, Fig. 5G). This increased excitability of *16p11.2*^+/-^ SST INs was underlain by a 6% hyperpolarisation of the voltage threshold (*p* = 0.0279 LME, Fig. 5I), and a tendency towards 17% reduced rheobase currents, albeit not significantly so (*p* = 0.1087 LME, Fig. 5H). All other physiological parameters were similar between genotypes (Additional file 1: Table S1).

To determine whether these *16p11.2* gene-dependent changes in SST IN function were cell type specific, we next performed recordings from CA1 PCs—the local ExNs (WT: n = 18 cells, N = 10 rats; *16p11.2*^+/-^: n = 18 cells, N = 9 rats). In response to the same hyper- to depolarising stimuli as used for SST INs, we observed no change in the either the number of action potentials produced by CA1 PCs in the *16p11.2*^+/-^ rats compared to WT (F_(5, 85)_ = 0.9185, P = 0.4731, 2-way ANOVA for current/genotype interaction, Fig. S6). Consistently, we observed no change in CA1 PC rheobase (*p* = 0.7098, LME, Fig. S6C), action potential threshold (*p* = 0.4116, LME, Fig. S6D), or any other parameter tested (Additional file 1: Table S1).

We and others have recently shown that cell-type specific changes to neuronal excitability may result from structural changes to the axon initial segment (AIS) in rodent models of neurological pathology [11, 15, 73, 77] reviewed in [28]. To determine if the *16p11.2*-dependent changes in SST IN excitability are due to altered AIS structure we performed correlated immunolabelling of SST and AnkyrinG in the hippocampus of WT (N = 5) and *16p11.2*^+/-^ (N = 5) rats. Immunofluorescent labelling for AnkyrinG reliably labelled the AIS of all neurons in the CA1 region, which were then identified as emerging from SST INs (Fig. 5J). Comparison of the AIS length of SST INs revealed that they were 11% longer in *16p11.2*^+/-^ rats, compared to WT littermates (*p* = 0.0064, LME, Fig. 5K). There was no change in the AIS length of putative CA1 PyrC (*p* = 0.0962, LME, Fig. 5L).

These data clearly highlight that loss of *16p11.2* genes, in a heterozygous manner, selectively impairs the function of SST INs, with no detectable change to local ExNs. The increased excitability of SST INs correlated well with the observed longer AIS of this cell type. Given the role for SST INs in gating spatial information flow in the hippocampus [40], this may plausibly explain the reported cognitive impairments in *16p11.2* microdeletion rodent models [69] and affected individuals [26, 34].

## Discussion

This study reveals that molecularly defined classes of INs in the foetal human cerebral cortex display enriched expression of *16p11.2* transcripts and a large number of monogenic autism risk factor transcripts suggesting they may represent a convergent target. This prompts the question of what is the identity of these cells? We examined human foetal cortical cells spanning the interval GW8-26 and found IN1, 3, 5, 8 cells are not present in the cerebral cortex before GW23 and then increase in numbers to GW26. As many INs have migrated into the cortex well before GW23 suggesting that IN1, 3, 5, 8 represent a relatively differentiated stage on the IN developmental trajectory. This is supported by pseudotime analysis and the enrichment of GO terms relating to synapse maturation and neurite formation in these cells. Transcriptomic similarity between foetal IN1, 3, 5, 8 and adult PV, SST, and VIP INs suggests these cells are destined to form a variety of IN cell types and that changes in their developmental trajectory caused by autism associated mutations may have far reaching consequences for the formation on inhibitory circuitry in the post-natal brain. In the current study we focussed on the stage of the IN development present in the developing cerebral cortex between GW8 and GW26 and this data does not include INs at earlier stages of their development as NPCs in the GE or as they migrate towards the cerebral cortex [33, 41], or at later post-natal stages. Indeed, many *16p11.2* transcripts are expressed by IN progenitors in the GE [46, 66, 84]. Studies on other types of NPC, both rodent cortical NPCs destined to become ExNs and hIPSC derived NPCs, show that the *16p11.2* microdeletion affects processes including gene expression, proliferation, and cell migration that occur before INs reach the cerebral cortex [19, 55, 56, 65, 76]. Overall it seems likely that the phenotypes of postnatal *16p11.2*^±^ INs may result in part from cumulative perturbations along their developmental trajectory although testing this hypothesis is well beyond the scope of the current study.

Heterozygous *16p11.2* microdeletion (*16p11.2*^+/-^) causes a twofold reduction in *16p11.2* transcript levels and differential expression of hundreds of transcripts outwith the *16p11.2* locus in several cellular contexts including in *16p11.2*^+/-^ rodent cerebral cortex and *16p11.2*^+/-^ human induced pluripotent stem cell (hIPSC) derived neurons [9, 22, 65, 70, 71, 76]. *16p11.2*^+/-^ IN1,3,5,8 cells and their rodent counterparts therefore likely experience reduced expression of *16p11.2* transcripts and differential expression of transcripts outwith the *16p11.2* locus which may contribute to the IN phenotypes prompting future investigation into how the *16p11.2* microdeletion impacts gene expression in developing INs.

Although the most striking enrichment of both monogenic and *16p11.2* risk factor transcripts was in INs we also observed enrichment in other cells, most notably in NPCs cluster ‘P6’. NPCs in the developing neocortex are destined to differentiate into ExNs and non-neuronal cell-types (eg astrocytes) suggesting these may be vulnerable and impact the development of ExNs. Consistent with this a variety of cell, molecular, anatomical, and functional phenotypes have been reported in in both rodent cortical and hIPSC derived *16p11.2*^+/-^ ExNs [22, 24, 45, 83].

We found that large numbers of monogenic autism risk factor transcripts are highly expressed in IN1, 3, 5, 8 INs, suggesting that their mutation may contribute to the aetiology of autism via alterations to IN development. Systematically testing this hypothesis for all of these risk factors presents a considerable challenge. However, for some including *ARID1B*, *DYRK1A*, *MECP2*, and *CNTNAP2, FMR1, and TSC2* there is already evidence that monogenic mutation causes abnormal numbers or physiological properties of INs in rodent models [1, 29, 36, 52, 68, 74, 78]. We also found *KCTD13*, *MAPK3*, and *MVP* transcripts expressed from the *16p11.2* locus are enriched in IN1,3,5,8. *KCTD13* modulates synaptic transmission by suppressing RHOA signalling via interaction with the ubiquitin ligase *CUL3*, itself an autism risk factor [24, 82]. *CUL3* is co-expressed with *KCTD13* in IN1, 3, 5, 8 cells suggesting a molecular mechanism for the *16p11.2* microdeletion to impact on cellular and synaptic function via perturbed RHOA signalling. Interestingly, the inhibition of RHOA pathway has been proposed as a treatment to restore cognition in *16p11.2* mouse models and ameliorates migratory phenotypes in hIPSC derived *16p11.2*^+/-^ neurons [42, 76]*. MAPK3* and *MVP* are both implicated in ERK signalling which impacts diverse cellular processes including cell proliferation, migration, and synaptic plasticity. Indeed, a mouse model of *16p11.2* microdeletion shows elevated ERK signalling leading to perturbed cortical development and autism-like phenotypes [55, 56], although the involvement of INs was not tested.

Our data shows the preferential expression of *16p11.2* genes in developing inhibitory INs over the developmental stages examined, which may lead to subsequent alterations to their functional properties. Indeed, electrophysiological recordings from identified SST INs in CA1 revealed that these cells were hyperexcitable which correlated with increased AIS length, despite no observed effect in neighbouring ExNs. We have previously shown that such changes in AIS length may in fact be causal to altered neuronal hyperexcitability in other monogenic models of autism [11], but this link in SST INs from the *16p11.2*^+/-^ rat remains unexplored. This data suggests that reduced *16p11.2* gene dosage leads to excessive signalling of SST INs in response to endogenous synaptic inputs. Such enhanced SST IN signalling in CA1 of the hippocampus has been shown to gate spatial inputs [40] and to impair spatial and fear learning [67], both of which are impaired in rodents lacking *16p11.2* genes [69, 72] and which may be rescued by GABA_B_ receptor activation [69]. Indeed, GABA_B_ receptors extremely tightly regulate the activity of hippocampal SST INs, preventing their control of the local circuit [10]. These studies provide a potential link between altered SST IN excitability in models of *16p11.2* microdeletion, which may explain impaired behavioural function. But further study is needed to determine the full mechanism by which such effects may interact with each other. Interestingly, in a mouse model of *16p11.2* duplication decreased synaptic inhibition was observed and corrected by overexpression of NPAS4 [62], which targets somatic and dendritic GABAergic synapse formation [8]. This study builds on a number of recent reports revealing how changes to intrinsic signalling of neurons, resulting from either direct or homeostatic alteration, in rodent models of neurodevelopmental and neuropsychiatric disorders leads to altered information processing in cortical circuits [1, 11, 15, 23, 31, 40, 77]. Our current study opens up further questions regarding the long-term functional consequences of SST IN hyperexcitability in cortical circuits, how this may directly affect behaviour, potentially leading to novel therapeutic interventions.

## Limitations

Whilst we provide evidence that developing INs in the human foetal cerebral cortex are potentially vulnerable to the *16p11.2* microdeletion the analysis of human gene expression data in the current study does not cover the full developmental trajectory of INs including before reach the cerebral cortex around GW16 or at foetal stages after GW26 and post-natally. Therefore we are unable to draw inferences about the potential vulnerabilities of INs to the *16p11.2* microdeletion at these stages from gene expression data. At present data covering the full IN developmental trajectory is not available, in part because INs migrate through the forebrain during their development so the different stages are not present in a single tissue dissection. Furthermore, our analysis of *16p11.2*^+/-^ rat phenotypes reported in the current study was restricted to 3-week old (P21) animals around the time of weaning and to a single interneuron cell-type, SST INs in CA1, while in human *16p11.2* microdeletion carriers symptoms likely involve multiple cell types in different areas and manifest in the first few years of life and on into adulthood.

## Conclusions

Our bioinformatic analysis of developing human foetal cerebral cortex gene expression suggests that the *16p11.2* microdeletion renders developing INs vulnerable to perturbation and this is supported in a *16p11.2* microdeletion rat model. This study adds to existing knowledge and prompts more in depth investigations of how monogenic autism risk factors and the *16p11.2* microdeletion act along IN development.

## Supplementary Information

**Additional file 1: Figure S1 (A)** *t*-SNE plot showing the distribution of foetal stages **(B)** table showing numbers of cells of each cardinal class at each foetal stage. **(C,D)** Transcriptomic relationships of IN clusters between Zhong’s clustering result (Zhong et al., 2018) and Yang’s clustering result (current study) of the same data. **(C)** Alluvial plot illustrating the relationship between Zhong’s clusters (left) and current Yang’s clusters (right). The size of the bars of each cluster is normalized to cell numbers. Cluster distinction is marked by different colours. **(D)** Comparison of transcriptomic similarity (Pearson’s correlation) between IN clusters defined in Zhong’s (y-axis) and Yang’s (x-axis) studies. **Figure S2** Pairwise comparison of the cluster similarity calculated by MetaNeighbor between the 21 cell clusters. AUROC scores represented as a heatmap where high similarity between clusters is coloured red and low similarity blue. Three plots are shown using different input gene sets **(A)** ~2000 highly variable gene transcripts between clusters. **(B)** the 83 high confidence and strong candidate (SFARI lists 1 and 2) monogenic autism risk factor transcripts. **(C)** the 27 *16p11.2* transcripts. **Figure S3 **Violin plots showing transcript levels in the 21 different clusters for **(A)** the 83 high confidence and strong candidate (SFARI lists 1 and 2) monogenic autism risk transcripts and **(B)** the 27 *16p11.2* transcripts. **Figure S4**. Characterisation of INs by pseudotime and gene ontology analysis. **(A-D)** pseudotime analysis. **(A) **UMAP layout visualizing the developmental trajectory of cortical INs by Monocle 3. **(B)** Distribution of cells in each IN clusters (IN 1-8) on pseodutime trajectory shown in A. **(C)** Distribution of cells among developmental stages (GW 08-26) on pseodutime trajectory shown in A. **(D)** Dynamic gene expression of *MEF2C*, *ADCY1*. *SYT4 and CAMK2* in IN clusters (IN1-8) along pseudotime trajectory, showing temporal specificity in the developing human cortical INs. **(E-G)** Gene ontology (GO) analysis. GO analysis transcripts expressed in IN5,8 (orange in **E**) versus all other cells (green in **E**) reveals **(F)** enrichment of GO terms associated with synaptic activity, maturation, and plasticity. **(G)** gradient plots of CAMK2, ADCY1 showing that these transcripts are most highly expressed in IN5,8. **Figure S5 **Violin plots showing transcript levels in mouse E18.5 and P20 cerebral cortex INs for **(A)** the 27 *16p11.2* transcripts and **(B)** the 83 high confidence and strong candidate (SFARI lists 1 and 2) monogenic autism risk transcripts. **Figure S6**: No change in total IN number in somatosensory cortex or hippocampus of the *16p11.2*^+/-^ rat. **(A)** Based on the expression of *Gad1 *mRNA, quantification of the total number of INs across the whole somatosensory column from WT (N=6) and *16p11.2*^+/-^ (N=6) rats. (**B**) No change in the relative ratio of *Gad1*-positive cells to total neurons (NeuN) was observed within the cortical column. (**C**) Expanded view of CA1 of the hippocampus from the same image as in Figure 5A, showing Gad1 in situ hybridisation (red), NeuN immunolabelling (green) and DAPI nuclei (blue). Regions used for cell counts in *str. pyramidale* (SP) and *str. oriens* (SO) are delineated with yellow lines. Scale bar: 200 µm. (**D**) Total number of *Gad1*-positive neurons measured in CA1 from WT (N=5) and *16p11.2*^+/-^ (N=5) rats. Statistics shown: ns – p>0.05 from Student’s 2-tailed t-test. **Figure S7:** No change in the excitability of CA1 PyrCs from the hippocampus of the *16p11.2*^+/-^ rat. **(A)** Representative action potential discharge in response to hyper- to depolarising current steps in CA1 PyrCs from WT (top) and 16p11.2^+/-^ rats (bottom). **(B)** Summary current-frequency plots from identified CA1 PyrCs from WT (N=10 rats) and *16p11.2*^+/-^ (N=9 rats). (**C**) Quantification of rheobase current in CA1 PyrCs from WT (n=18 cells) and *16p11.2*^+/-^ (n=18 cells) rats. (**D**) Voltage threshold of the first action potential elicited at rheobase for the same cells. Statistics shown: ns – p>0.05, from Linear Mixed Effects modelling. **Figure S8:** Individual example confocal images showing the start and end points of the AIS with respect to SSt INs. Representative individual images from a confocal Z-stack displaying immunolabelling for AnkyrinG (magenta), SSt (green), and merged; from WT (upper) and *16p11.2*^+/-^ rats (lower). The starting position of the AIS is identified in all images (white arrows). **Table S1. **electrophysiological parameters for Sst INs and CA1 pyramidal cells in P21 wild-type and 16p11.2^+/-^ rats.

## Data Availability

The datasets generated during the current study are available from the corresponding author on reasonable request.

## Abbreviations

ACSF: Artificial cerebrospinal fluid
AIS: Axon initial segment
AUROC: Area under the receiver operating characteristic
ASC: Autism spectrum condition
CCA: Canonical correlation analysis
CNV: Copy number variation
DEG: Differentially expressed gene
E/I: Excitatory/inhibitory
EPSC: Excitatory postsynaptic current
ExN: Excitatory neuron
GABA: Gamma-aminobutyric acid
GE: Ganglionic eminence
GO: Gene ontology
GW: Gestational week
hIPSC: Human induced pluripotent stem cell
HVG: Highly variable gene
ID: Intellectual disability
IN: Interneuron
KNN: *k*-Nearest neighbors
LME: Linear mixed effect
NPC: Neural progenitor cell
OPC: Oligodendrocyte precursor
P21: Post-natal day 21
PBS: Phosphate buffered saline
PC: Pyramidal cell
PCA: Principal component analysis
scRNA-seq: Single cell RNA sequencing
SEM: Standard error of the mean
SO: Str. Oriens
SP: St. pyramidale
SST: Somatostatin
tSNE: T-distributed stochastic neighbour embedding
WT: Wild-type
