# Supplementary material for: Identifying foetal forebrain interneurons as a target for monogenic autism risk factors and the polygenic 16p11.2 microdeletion
Source: BMC Neurosci. 2023 Jan 19;24:5. doi: 10.1186/s12868-022-00771-3 (PMC9850541; doi:10.1186/s12868-022-00771-3)
Supplement: Supplementary file 1 — Additional file 1: Figure S1 (A) t-SNE plot showing the distribution of foetal stages (B) table showing numbers of cells of each cardinal class at each foetal stage. (C,D) Transcriptomic relationships of IN clusters between Zhong’s clustering result (Zhong et al., 2018) and Yang’s clustering result (current study) of the same data. (C) Alluvial plot illustrating the relationship between Zhong’s clusters (left) and current Yang’s clusters (right). The size of the bars of each cluster is normalized to cell numbers. Cluster distinction is marked by different colours. (D) Comparison of transcriptomic similarity (Pearson’s correlation) between IN clusters defined in Zhong’s (y-axis) and Yang’s (x-axis) studies. Figure S2 Pairwise comparison of the cluster similarity calculated by MetaNeighbor between the 21 cell clusters. AUROC scores represented as a heatmap where high similarity between clusters is coloured red and low similarity blue. Three plots are shown using different input gene sets (A) ~2000 highly variable gene transcripts between clusters. (B) the 83 high confidence and strong candidate (SFARI lists 1 and 2) monogenic autism risk factor transcripts. (C) the 27 16p11.2 transcripts. Figure S3 Violin plots showing transcript levels in the 21 different clusters for (A) the 83 high confidence and strong candidate (SFARI lists 1 and 2) monogenic autism risk transcripts and (B) the 27 16p11.2 transcripts. Figure S4. Characterisation of INs by pseudotime and gene ontology analysis. (A-D) pseudotime analysis. (A) UMAP layout visualizing the developmental trajectory of cortical INs by Monocle 3. (B) Distribution of cells in each IN clusters (IN 1-8) on pseodutime trajectory shown in A. (C) Distribution of cells among developmental stages (GW 08-26) on pseodutime trajectory shown in A. (D) Dynamic gene expression of MEF2C, ADCY1. SYT4 and CAMK2 in IN clusters (IN1-8) along pseudotime trajectory, showing temporal specificity in the developing human cortical INs. (E-G) Gen [file 12868_2022_771_MOESM1_ESM.docx]

**Additional file**

**
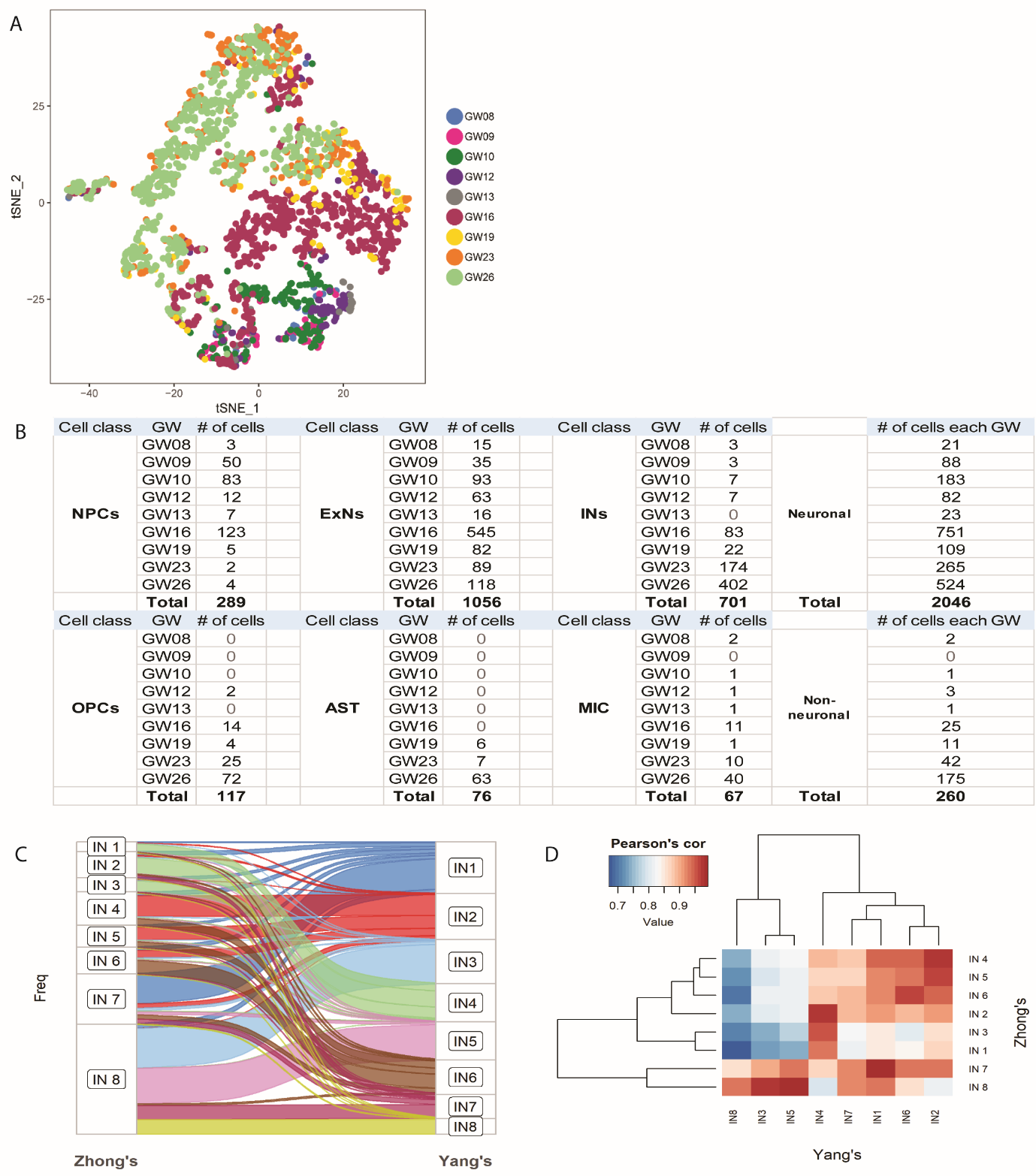
**

**Figure S1 (A)** *t*-SNE plot showing the distribution of foetal stages **(B)** table showing numbers of cells of each cardinal class at each foetal stage. **(C,D)** Transcriptomic relationships of IN clusters between Zhong’s clustering result (Zhong et al., 2018) and Yang’s clustering result (current study) of the same data. **(C)** Alluvial plot illustrating the relationship between Zhong’s clusters (left) and current Yang’s clusters (right). The size of the bars of each cluster is normalized to cell numbers. Cluster distinction is marked by different colours. **(D)** Comparison of transcriptomic similarity (Pearson’s correlation) between IN clusters defined in Zhong’s (y-axis) and Yang’s (x-axis) studies.

**
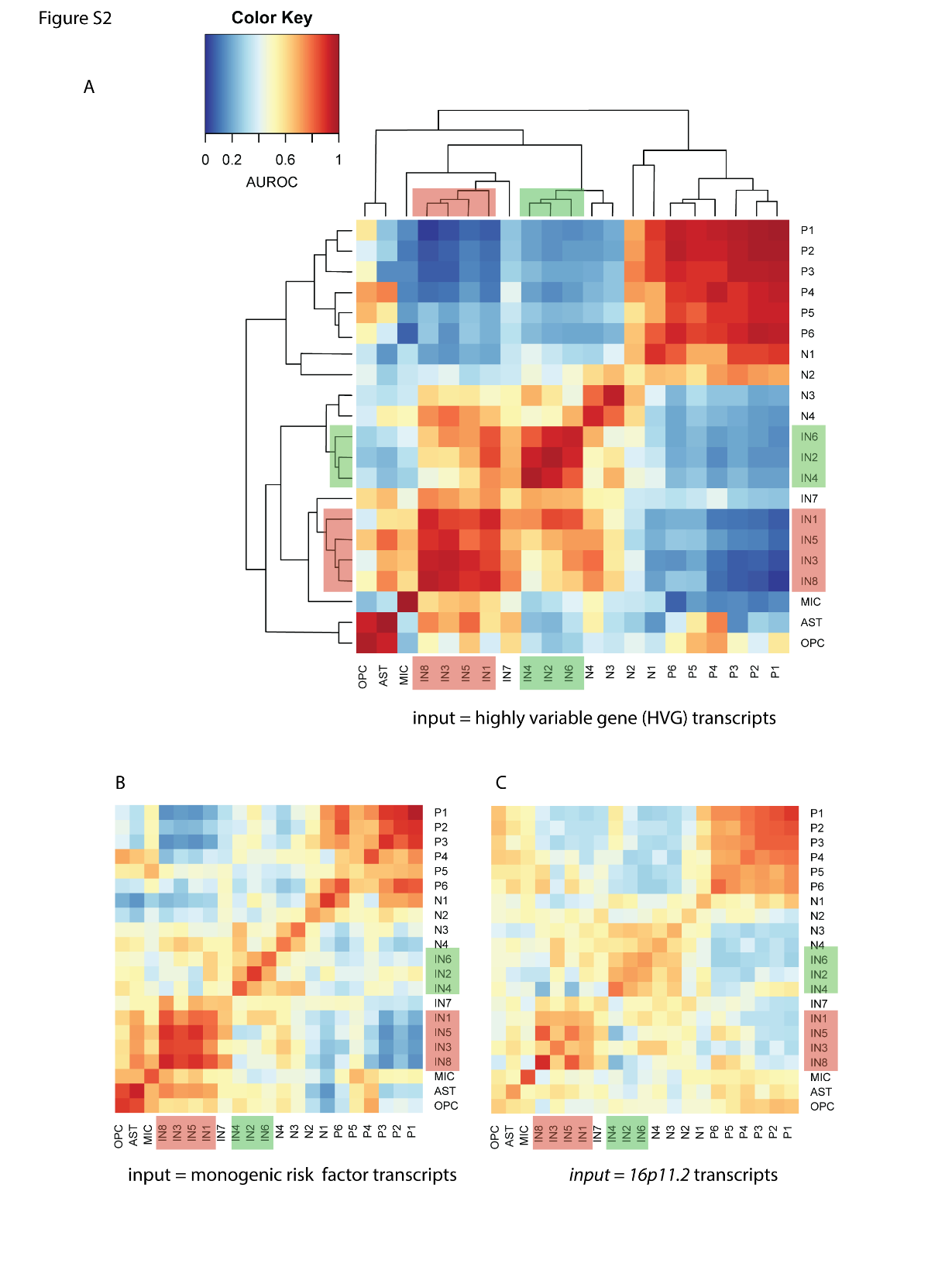
**

**Figure S2** Pairwise comparison of the cluster similarity calculated by MetaNeighbor between the 21 cell clusters. AUROC scores represented as a heatmap where high similarity between clusters is coloured red and low similarity blue. Three plots are shown using different input gene sets **(A)** ~2000 highly variable gene transcripts between clusters. **(B)** the 83 high confidence and strong candidate (SFARI lists 1 and 2) monogenic autism risk factor transcripts. **(C)** the 27 *16p11.2* transcripts.

**
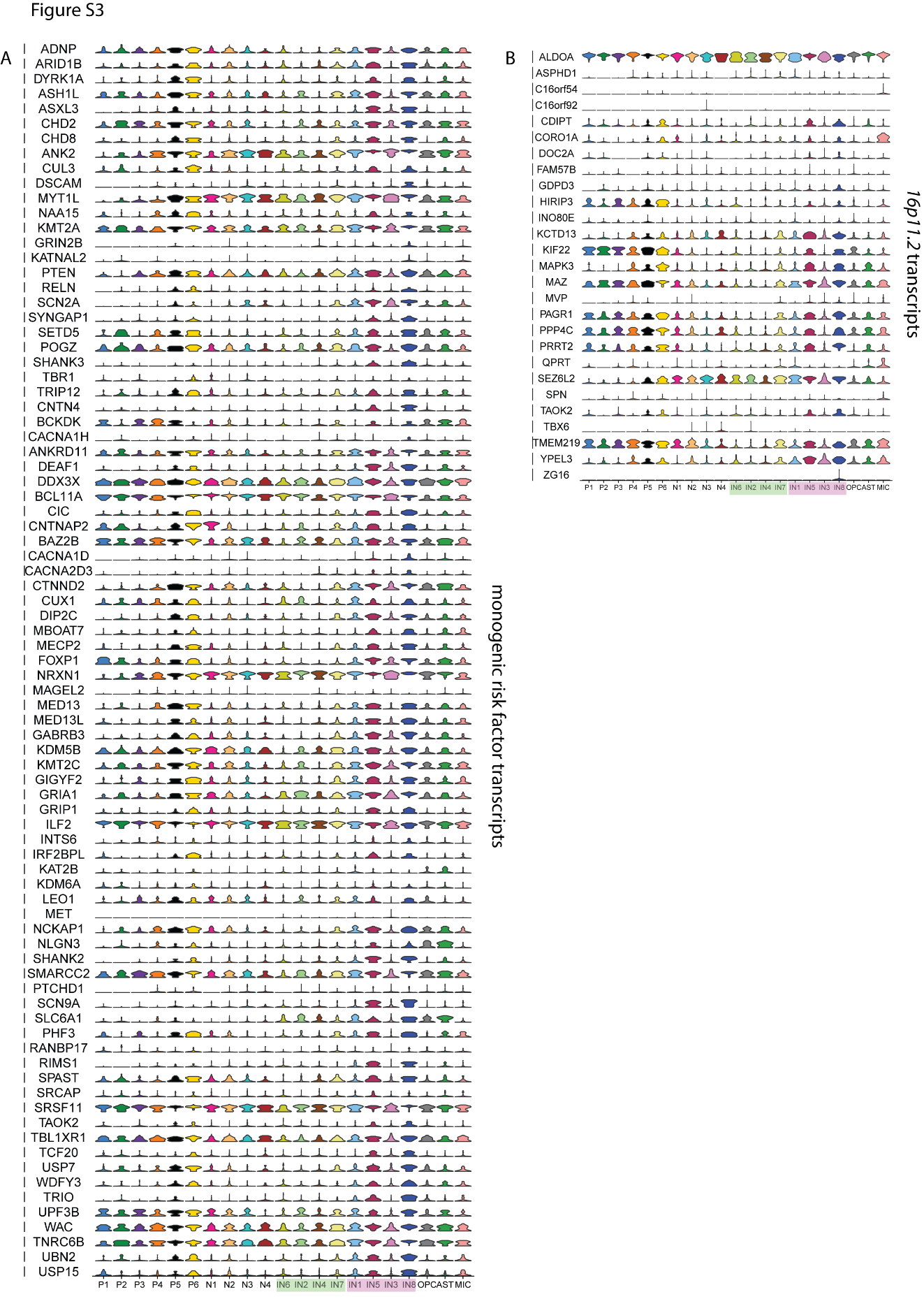
Figure S3** Violin plots showing transcript levels in the 21 different clusters for **(A)** the 83 high confidence and strong candidate (SFARI lists 1 and 2) monogenic autism risk transcripts and **(B)** the 27 *16p11.2* transcripts.

**
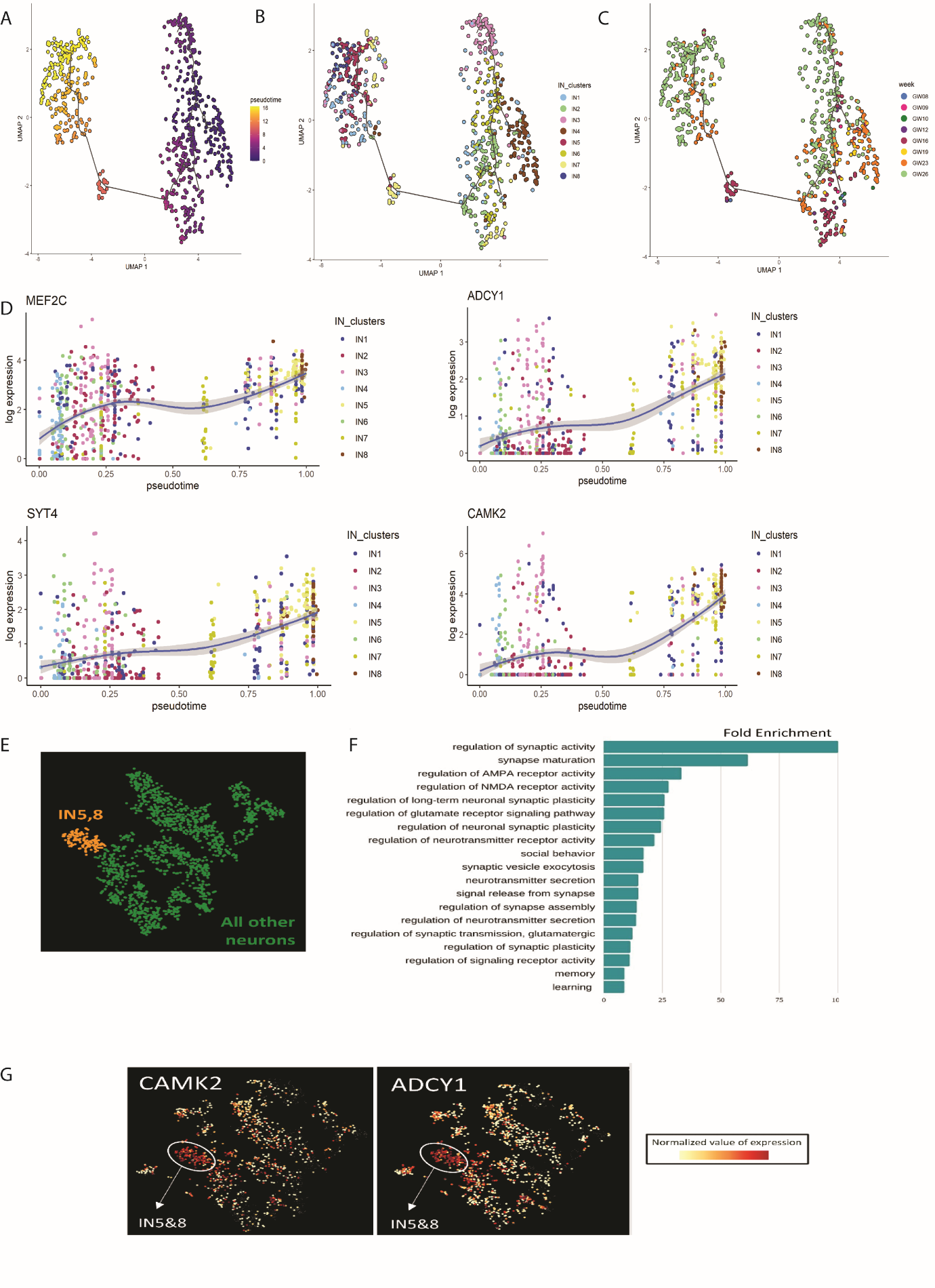
**

**Figure S4**. Characterisation of INs by pseudotime and gene ontology analysis. **(A-E)** pseudotime analysis. **(A)** UMAP layout visualizing the developmental trajectory of cortical INs by Monocle 3. **(B)** Distribution of cells in each IN clusters (IN 1-8) on pseodutime trajectory shown in A. **(C)** Distribution of cells among developmental stages (GW 08-26) on pseodutime trajectory shown in A. **(D,E,F)** Dynamic gene expression of *MEF2C* **(D)**, *ADCY1* **(E)** and *SYT4* **(F)** in IN clusters (IN1-8) along pseudotime trajectory, showing temporal specificity in the developing human cortical INs. **(E-G)** Gene ontology (GO) analysis. GO analysis transcripts expressed in IN5,8 (orange in **E**) versus all other cells (green in **E**) reveals **(F)** enrichment of GO terms associated with synaptic activity, maturation, and plasticity. **(G)** gradient plots of CAMK2, ADCY1 showing that these transcripts are most highly expressed in IN5,8.


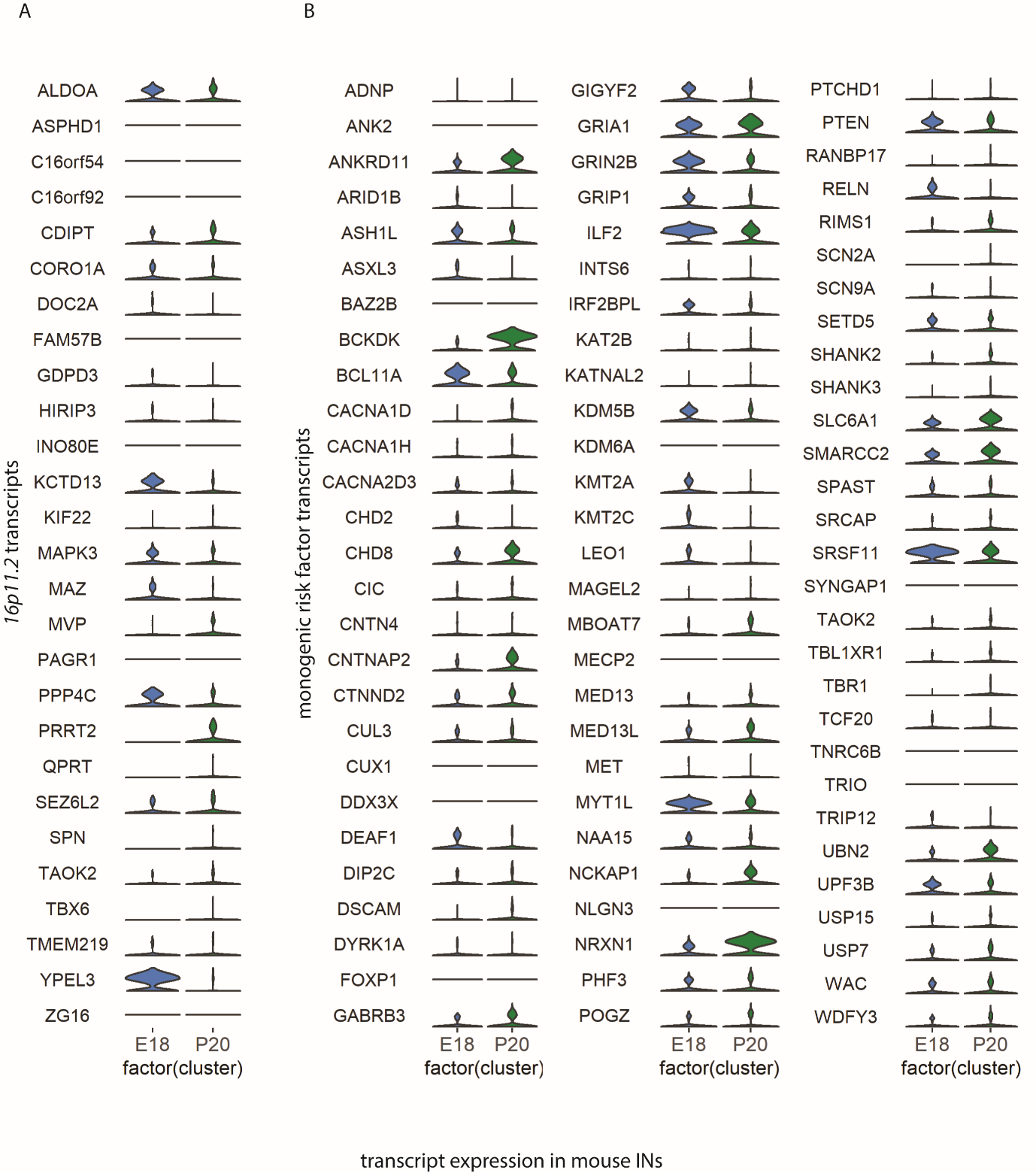


**Figure S5** Violin plots showing transcript levels in mouse E18.5 and P20 cerebral cortex INs for **(A)** the 27 *16p11.2* transcripts and **(B)** the 83 high confidence and strong candidate (SFARI lists 1 and 2) monogenic autism risk transcripts.


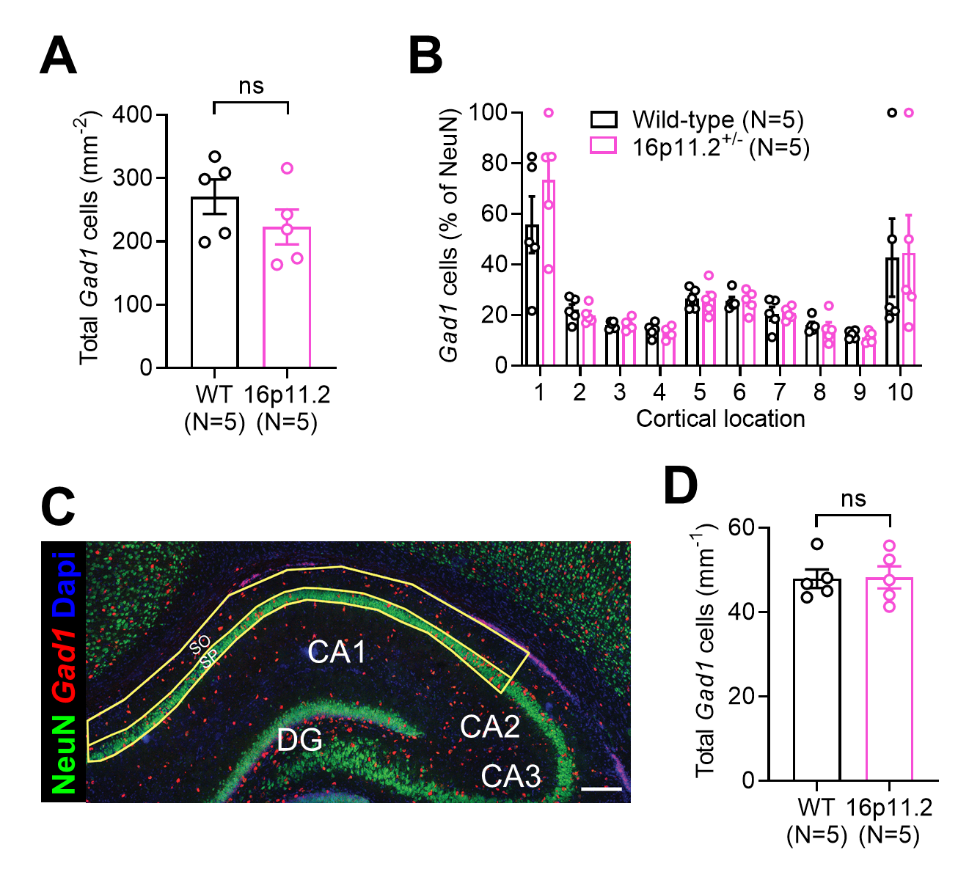


**Figure S6: No change in total IN number in somatosensory cortex or hippocampus of the *16p11.2*^+/-^ rat. (A)** Based on the expression of *Gad1* mRNA, quantification of the total number of INs across the whole somatosensory column from WT (N=6) and *16p11.2*^+/-^ (N=6) rats. (**B**) No change in the relative ratio of *Gad1*-positive cells to total neurons (NeuN) was observed within the cortical column. (**C**) Expanded view of CA1 of the hippocampus from the same image as in Figure 5A, showing Gad1 in situ hybridisation (red), NeuN immunolabelling (green) and DAPI nuclei (blue). Regions used for cell counts in *str. pyramidale* (SP) and *str. oriens* (SO) are delineated with yellow lines. Scale bar: 200 µm. (**D**) Total number of *Gad1*-positive neurons measured in CA1 from WT (N=5) and *16p11.2*^+/-^ (N=5) rats. Statistics shown: ns – p>0.05 from Student’s 2-tailed t-test.


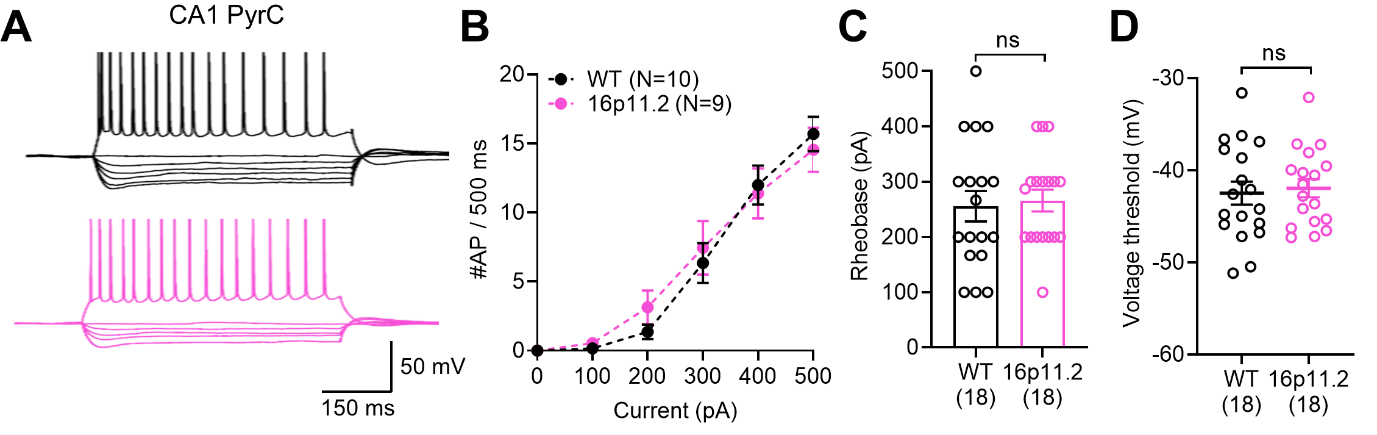


**Figure S7: No change in the excitability of CA1 PyrCs from the hippocampus of the *16p11.2*^+/-^ rat. (A)** Representative action potential discharge in response to hyper- to depolarising current steps in CA1 PyrCs from WT (top) and 16p11.2^+/-^ rats (bottom). **(B)** Summary current-frequency plots from identified CA1 PyrCs from WT (N=10 rats) and *16p11.2*^+/-^ (N=9 rats). (**C**) Quantification of rheobase current in CA1 PyrCs from WT (n=18 cells) and *16p11.2*^+/-^ (n=18 cells) rats. (**D**) Voltage threshold of the first action potential elicited at rheobase for the same cells. Statistics shown: ns – p>0.05, from Linear Mixed Effects modelling.

**Figure S8:** Individual example confocal images showing the start and end points of the AIS with respect to SSt INs. Representative individual images from a confocal Z-stack displaying immunolabelling for AnkyrinG (magenta), SSt (green), and merged; from WT (upper) and *16p11.2*^+/-^ rats (lower). The starting position of the AIS is identified in all images (white arrows).


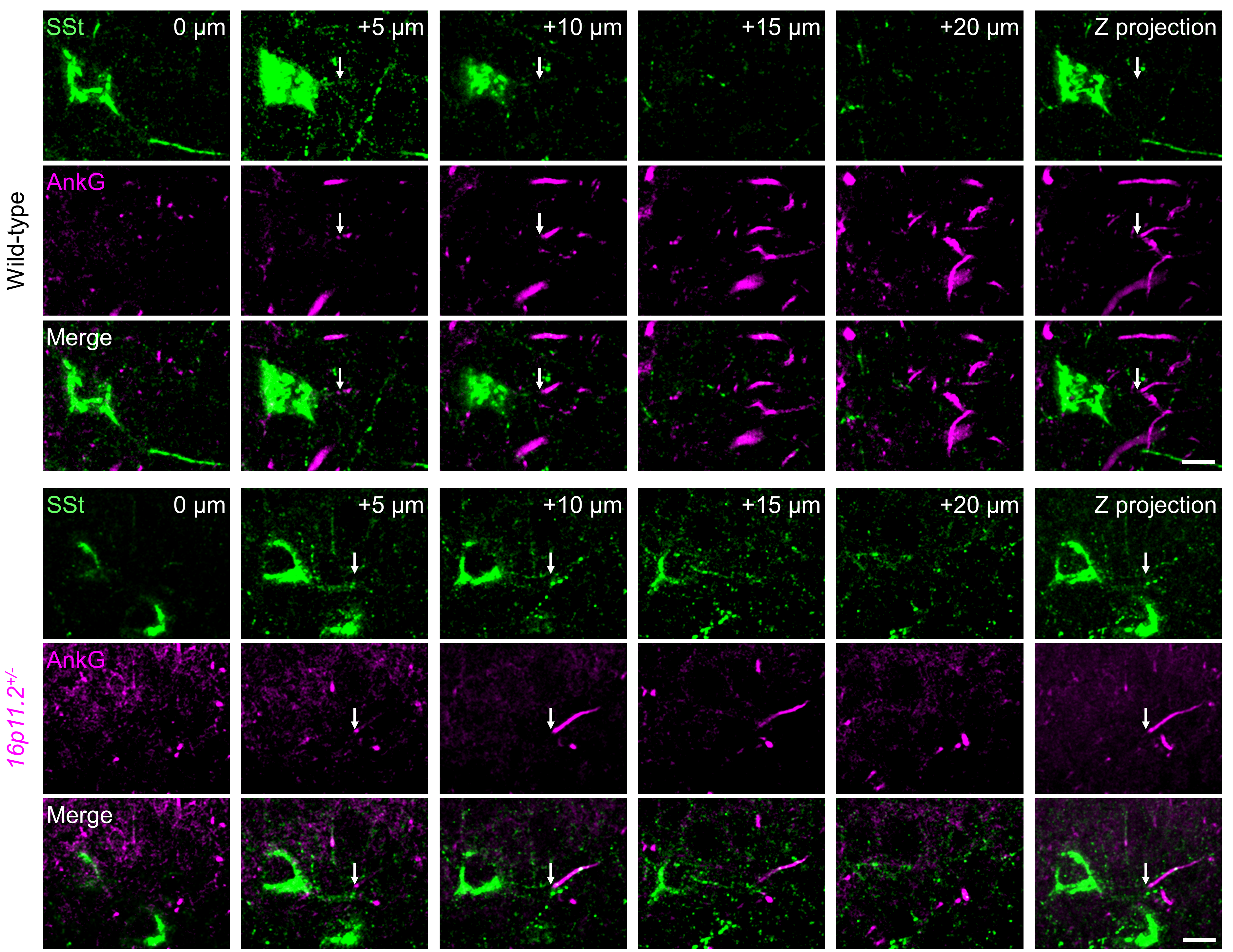


**Additional file tables**

**Table S1**

|  | **SSt INs** | | | **CA1 pyramidal cells** | | |
| --- | --- | --- | --- | --- | --- | --- |
| Parameter tested | Wild-type | 16p11.2^+/-^ | *p* | Wild-type | 16p11.2^+/-^ | *p* |
|  |  |  |  |  |  |  |
| n (N) | 30 (15) | 18 (13) |  | 18 (10) | 18(9) |  |
|  |  |  |  |  |  |  |
| Resting membrane potential (mV) | -56.6 ± 1.5 | -60.9 ± 1.8 | 0.25 | -64.6 ± 1.9 | -65.3 ± 1.1 | 0.88 |
| Input resistance (MΩ) | 204 ± 22 | 220 ± 26 | 0.65 | 113 ± 15 | 105 ± 18 | 0.73 |
| Membrane time-constant (ms) | 34.5 ± 5.3 | 34.9 ± 6.3 | 0.72 | 24.2 ± 1.7 | 23.0 ± 2.3 | 0.74 |
| Membrane capacitance (pF) | 162 ± 11 | 152 ± 16 | 0.30 | 239 ± 17 | 245 ± 16 | 0.97 |
|  |  |  |  |  |  |  |
| Voltage sag (mV) | 18.2 ± 1.7 | 17.9 ± 2.0 | 0.50 | 8.4 ± 0.6 | 9.4 ± 1.2 | 0.43 |
| Sag (% of maximum) | 32.9 ± 2.2 | 36.0 ± 3.7 | 0.76 | 23.9 ± 1.6 | 25.1 ± 1.5 | 0.65 |
|  |  |  |  |  |  |  |
| Rheobase (pA) | 233 ± 22 | 194 ± 28 | 0.11 | 256 ± 28 | 266 ± 20 | 0.71 |
| Voltage threshold (mV) | -39.6 ± 0.7 | -42.1 ± 0.9 | **0.03** | -42.4 ± 1.3 | -41.9 ± 1.0 | 0.41 |
| AP amplitude (mV) | 98.3 ± 3.2 | 97.7 ± 3.3 | 0.64 | 122.5 ± 1.8 | 119.8 ± 2.9 | 0.61 |
| AP 20-80% rise-time (ms) | 0.17 ± 0.01 | 0.16 ± 0.01 | 0.38 | 0.15 ± 0.01 | 0.16 ± 0.01 | 0.87 |
| AP half-height duration (ms) | 0.62 ± 0.02 | 0.61 ± 0.04 | 0.81 | 1.00 ± 0.02 | 0.98 ± 0.03 | 0.60 |
| AP max. rise-rate (mV.ms^‑1^) | 276 ± 18 | 310 ± 22 | 0.48 | 455 ± 19 | 443 ± 29 | 0.96 |
| AP max. decay-rate (mV.ms^‑1^) | 124 ± 7 | 139 ± 13 | 0.53 | 77 ± 1 | 79 ± 4 | 0.64 |
| Peak firing (Hz) | 54.8 ± 5.0 | 71.6 ± 7.9 | **0.02** | 30.3 ± 3.1 | 31.4 ± 1.8 | 0.70 |
